# Health system quality and COVID-19 vaccination: a cross-sectional analysis in 14 countries

**DOI:** 10.1016/S2214-109X(23)00490-4

**Published:** 2023-12-11

**Authors:** Catherine Arsenault, Todd P Lewis, Neena R Kapoor, Emelda A Okiro, Hannah H Leslie, Patrizio Armeni, Prashant Jarhyan, Svetlana V Doubova, Katherine D Wright, Amit Aryal, Sengchanh Kounnavong, Sailesh Mohan, Emily Odipo, Hwa-Young Lee, Jeonghyun Shin, Wondimu Ayele, Jesús Medina-Ranilla, Laura Espinoza-Pajuelo, Anagaw Derseh Mebratie, Ezequiel García Elorrio, Agustina Mazzoni, Juhwan Oh, Gillian K SteelFisher, Rosanna Tarricone, Margaret E Kruk

**Affiliations:** a Department of Global Health, Milken Institute School of Public Health, The George Washington University, Washington DC, USA; b Department of Global Health and Population, Harvard T H Chan School of Public Health, Boston, MA, USA; c Department of Health Policy and Management, Harvard T H Chan School of Public Health, Boston, MA, USA; d Population & Health Impact Surveillance Group, KEMRI Wellcome Trust Research Programme, Nairobi, Kenya; e Division of Prevention Science, Department of Medicine, University of California San Francisco, San Francisco, CA, USA; f CERGAS SDA Bocconi School of Management, Bocconi University, Milan, Italy; g Public Health Foundation of India, Gurgaon, Haryana, India; h Epidemiology and Health Services Research Unit CMN Siglo XXI, Mexican Institute of Social Security, Mexico City, Mexico; i Swiss Tropical and Public Health Institute and University of Basel, Basel, Switzerland; j Lao Public Health Association, Ministry of Health, Vientiane, Laos; k Graduate School of Public Health and Healthcare Management, The Catholic University of Korea, Seoul, South Korea; l Department of Medicine, Seoul National University College of Medicine, Seoul, South Korea; m School of Public Health, Addis Ababa University, Addis Ababa, Ethiopia; n Faculty of Public Health and Administration, Epidemiology Department, Universidad Peruana Cayetano Heredia, Lima, Peru; o Institute for Clinical Effectiveness and Health Policy, Buenos Aires, Argentina

## Abstract

The social and behavioural determinants of COVID-19 vaccination have been described previously. However, little is known about how vaccinated people use and rate their health system. We used surveys conducted in 14 countries to study the health system correlates of COVID-19 vaccination. Country-specific logistic regression models were adjusted for respondent age, education, income, chronic illness, history of COVID-19, urban residence, and minority ethnic, racial, or linguistic group. Estimates were summarised across countries using random effects meta-analysis. Vaccination coverage with at least two or three doses ranged from 29% in India to 85% in Peru. Greater health-care use, having a regular and high-quality provider, and receiving other preventive health services were positively associated with vaccination. Confidence in the health system and government also increased the odds of vaccination. By contrast, having unmet health-care needs or experiencing discrimination or a medical mistake decreased the odds of vaccination. Associations between health system predictors and vaccination tended to be stronger in high-income countries and in countries with the most COVID-19-related deaths. Access to quality health systems might affect vaccine decisions. Building strong primary care systems and ensuring a baseline level of quality that is affordable for all should be central to pandemic preparedness strategies.

This is the sixth in a **Series** of six papers about the People's Voice Survey on Health System Performance. All papers in the Series are available at www.thelancet.com/series/peoples-voice-survey

## Background

The development of multiple vaccines against COVID-19 within 1 year of the virus's emergence was unprecedented. However, the global effort to immunise a critical mass of the world's population has been challenged by the rise in COVID-19 vaccine misinformation and hesitancy.^1^, ^2^ Inequitable distribution of vaccines globally also affected uptake in lower-income countries.^3^ By the end of 2022, an estimated 13·2 billion doses had been administered.^4^ Nonetheless, a large fraction of the world's population remains unvaccinated or underimmunised against COVID-19.

The demographic drivers of COVID-19 vaccine uptake (including booster uptake) have been described across multiple contexts.^1^, ^5^, ^6^, ^7^ Lower education, lower income, younger age, female gender, and living in a rural area have been associated with poorer acceptance.^1^, ^5^, ^6^ COVID-19 vaccine uptake was initially also lower among minority racial and ethnic groups but appears to have increased over time.^8^ In addition, mistrust of government and health authorities has been linked to poorer vaccine uptake,^1^, ^9^ and several studies have shown vaccination hesitancy to be a key driver of poor uptake.^2^

Despite these predisposing factors, high-quality health systems could have the potential to improve vaccination rates. Research has shown that high-quality care, particularly that perceived as such by the patient, can affect behaviours such as retention in care and adherence to treatments.^10^, ^11^ More frequent exposure to good-quality care (ie, care that is respectful, continuous, evidence-based, and person-centred) might also improve confidence in health-care interventions such as COVID-19 vaccination.

Nonetheless, there is currently little evidence on the patterns of health-care use among vaccinated individuals. For example, little is known about how vaccinated people interact with the health system, whether and how they use other health services, and how they rate the quality of care they receive. Understanding how vaccinated individuals use and rate health care and their health system could help in the design of targeted strategies to improve vaccine uptake.

**Key messages**

• Greater health-care use in the last year, use of other preventive health services, and having a high-quality usual provider increased the odds of COVID-19 vaccination (adjusted odds ratios [aORs] pooled by random effects meta-analysis across the 14 countries ranged from 1·15 to 1·80)
• A sense of health security (being confident of being able to access and afford quality care) and other measures of confidence in the government and the health system also increased COVID-19 vaccination (aORs pooled by random effects meta-analysis across the 14 countries ranged from 1·28 to 1·42)
• Having experienced discrimination in the health system, medical errors, and unmet health-care needs decreased the odds of vaccination (aORs pooled by random effects meta-analysis across the 14 countries ranged from 0·83 to 0·72)
• Associations were stronger in high-income countries, possibly reflecting a greater degree of patient activation, higher expectations, and the more widespread availability of COVID-19 vaccines
• Associations were also stronger in countries with a greater number of COVID-19-related deaths, indicating that health systems might play a bigger role in affecting vaccine decisions when countries are faced with more severe crises
• Recent experiences in the health system appear to affect COVID-19 vaccine decisions; our findings indicate the importance of increasing access to affordable, high-quality health care as a strategy to promote vaccine uptake during health emergencies

In this study, we aimed to identify the health system correlates of COVID-19 vaccination in 14 countries. In addition to demographic factors, we focused on health-care use, user-reported health system competence, perceived quality, user experience, and confidence in the health system and the government.

## Data source

This analysis used the People's Voice Survey—a cross-sectional instrument developed by the Quality Evidence for Health System transformation (QuEST) Network to study people's perspectives of health care and their health system. The survey was administered from May, 2022, to April, 2023, in 14 countries, including four low-income or lower-middle-income countries (Ethiopia, India, Kenya, and Laos), five upper-middle-income countries (Argentina, Colombia, Mexico, Peru, and South Africa), and five high-income countries (Italy, South Korea, the UK, the USA, and Uruguay). Country characteristics and survey methods are described in appendix 1. The survey was administered primarily by telephone and online in South Korea, the USA, and the UK. Samples were nationally representative of the adult population in each country except in Argentina, where the sample was representative of the adult population of the province of Mendoza only.

## Measures

In this analysis, we focused on the individuals who had received the most doses of COVID-19 vaccine in each country. At the time of the survey, most countries recommended a minimum of two COVID-19 vaccine doses. Nonetheless, recommendations varied for different risk groups (eg, according to age, comorbidities, pregnancy, or work setting) or according to the type of first dose or previous boosters received. Given these nuances in recommendations, and to account for important differences in vaccine supply across countries, we created two groups of countries for the main analysis. The first group (Ethiopia, Kenya, and South Africa) consisted of countries that secured less than two COVID-19 vaccine doses per population as of Aug 31, 2022 (appendix 2 p 2).^12^ In these three countries, we defined the most-vaccinated individuals as those with at least two doses of a COVID-19 vaccine. In all other countries, with greater vaccine supply (ranging from 2·01 doses per capita in India to 7·48 doses per capita in Italy), we defined the most-vaccinated individuals as those who received at least three doses of a COVID-19 vaccine.^12^ Depending on the vaccine type (which was not assessed in our survey), three doses would include those with a complete primary series and a booster, or a primary series and two booster doses.^13^

Our selection of health system quality measures was guided by the high-quality health system framework of the *Lancet Global Health* Commission on high quality health systems in the SDG Era.^14^ According to this framework, measurement of quality should focus on competent care and systems, user experience, and confidence in the health system. The predictors of interest were divided into six groups: the first included health-care use levels, the second included measures of health system competence, and the third included measures of perceived quality of care and user experience; we also independently investigated three measures of confidence in the health system and the government.

Health-care use was assessed according to the number of health-care visits reported in the last year, including in-person visits, telemedicine, and home visits; respondents were divided into those with no health-care visits, one or two visits, three or four visits, or five or more visits. Health system competence was assessed using three measures: whether the respondent had a usual source of care (ie, a regular provider or health facility from which they received most of their health care), had received at least three other recommended preventive health services, and had unmet health-care needs in the last year. The preventive health services assessed included a blood pressure check, blood sugar test, blood cholesterol test, eye examination, and dental examination.

The third group of predictors included three measures of perceived quality of care and user experience. These were available only for respondents with a usual source of care and at least one visit in the last year. The first measure, the perceived quality of the usual facility or provider, was measured on a five-point Likert scale (excellent, very good, good, fair, or poor quality). We compared those who rated the quality as excellent or very good with those who rated quality as good, fair, or poor. User experience was assessed by asking respondents to report whether they had experienced discrimination in the health system and whether they believed a medical mistake was made in their care.

We also separately assessed three measures of confidence in the health system and government. The first measure of confidence reflected the respondent's sense of health security—ie, their confidence in being able to get and afford quality care if they became ill. The second measure reflected confidence in the government's responsiveness to public input when making decisions about the health system; we compared those who were somewhat or very confident with those who were not too confident or not at all confident. Finally, respondents were asked to rate their government's management of the COVID-19 pandemic using a five-point Likert scale. Responses were dichotomised as above. Survey questions for the 12 measures of health system use and quality are available in appendix 2 (pp 4–5).

We included a total of eight demographic and health characteristics that might affect COVID-19 vaccination. These characteristics were respondent age, whether respondents had a chronic illness or a history of COVID-19, education, income, gender, and urban residence. In Ethiopia, Kenya, Laos, Mexico, Peru, South Africa, the USA, and the UK, we also asked whether the respondent belonged to a minority ethnic, racial, or linguistic group. Country-specific definitions of minority groups are in appendix 2 (p 6). For comparison across countries, education was summarised according to post-secondary education attendance. Income was based on self-reported annual or monthly household or individual income and divided into within-country tertiles.

## Analysis

We used multivariable logistic regression models to investigate associations between the measures of health system use and quality and COVID-19 vaccination (at least two or three doses). We used six separate regression models, conducted separately in each country. The first model assessed health-care use levels over the past year, the second included the three measures of health system competence, and the third included perceived quality and user experience among those with a usual source of care. Finally, because the three confidence measures were strongly correlated, we assessed their association with COVID-19 vaccination in three separate additional models. All six regression models included robust standard errors and were adjusted for the eight demographic and health-related factors described earlier. Potential multicollinearity between the independent variables and the eight covariates was assessed using variance inflation factors.

To summarise results from the six different regression models across countries, we pooled the estimates across all countries using inverse-variance-weighted random-effects meta-analyses.^15^ This approach combines country-specific estimates with the DerSimonian and Laird inverse-variance method and does not assume a common homogeneous estimate across all countries. The pooled estimates, therefore, assign more weight to the country-specific estimates that are more precise. We also pooled the estimates across country groups, including countries grouped by income (low-income and lower-middle-income countries, upper-middle-income countries, and high-income countries) and countries grouped by numbers of COVID-19-related deaths per million population at the start of the survey. Finally, given the complexities in defining up-to-date vaccination across countries, we repeated the analyses using at least two doses of a COVID-19 vaccine as the outcome in all countries.

## Findings on health-care use and perceived quality

**Figure 1:**
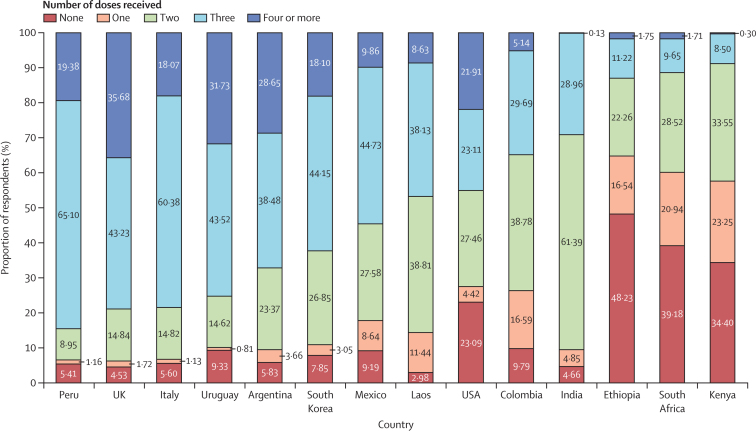
Reported number of COVID-19 vaccine doses received by country Countries are ordered by the proportion of respondents who received three or more doses of a COVID-19 vaccine. Samples are representative of the adult population in each country except Argentina, where respondents represent the province of Mendoza only. All estimates include sampling weights.

Our analysis included a total of 23 230 respondents from 14 countries. The proportion of respondents who had been vaccinated with at least two doses of a COVID-19 vaccine was 35·2% in Ethiopia, 39·9% in South Africa, and 42·5% in Kenya (figure 1). In the other countries, vaccination with at least three doses varied from 29·1% in India to 84·5% in Peru. Respondent characteristics are in appendix 2 (p 7).

Health system use and quality by country is shown in table 1. Health-care use was highest in Uruguay and the USA, with, on average, 7·5 and 7·3 health-care visits per respondent, respectively, in the year leading up to the survey. Between 48·7% (India) and 93·8% (Uruguay) of respondents reported having a regular health facility or provider from whom they received most of their health care (such as a primary care provider). Receipt of preventive health services was lowest in Ethiopia and Kenya and highest in South Korea and the USA. On average, across all countries, 14·2% of respondents had unmet health-care needs. The proportion of respondents with unmet health-care needs was highest in Peru, where 25·8% of respondents reported not seeking health care despite needing medical attention at some point in the last year, and lowest in South Korea (5·9%) and Italy (6·2%). Among those with a usual source of care, respondents from the USA and Argentina were the most likely to rate the quality as very good or excellent. On average, across all countries, 9·1% of respondents reported being treated unfairly or discriminated against in the health system in the past year, and 9·7% reported a medical mistake. Across the three confidence measures, confidence was highest in Laos, India, and Ethiopia and lowest in Peru, Colombia, and the UK.

**Table 1:** Health system use and quality ratings by People's Voice Survey respondents, by country

	**Argentina (n=1190)**	**Colombia (n=1237)**	**Ethiopia (n=2779)**	**India (n=2004)**	**Italy (n=1001)**	**Kenya (n=2305)**	**South Korea (n=2000)**	**Laos (n=2007)**	**Mexico (n=1002)**	**Peru (n=1255)**	**South Africa (n=2036)**	**UK (n=1677)**	**Uruguay (n=1237)**	**USA (n=1500)**
**Health-care use in the past year**														
One or two visits	20·3%	27·6%	32·4%	19·9%	27·2%	30·6%	22·3%	36·4%	27·7%	27·7%	26·2%	18·6%	21·6%	20·0%
Three to four visits	22·8%	24·9%	20·4%	18·2%	23·0%	23·8%	25·9%	18·6%	17·7%	21·4%	21·6%	27·0%	18·5%	25·6%
Five or more visits	45·4%	34·1%	11·5%	24·1%	30·0%	24·5%	46·6%	12·6%	34·0%	33·5%	30·4%	43·6%	50·5%	47·6%
**Health system competence**														
Had a usual source of care	83·5%	78·2%	71·6%	48·7%	74·7%	69·3%	62·9%	88·5%	81·9%	76·3%	67·5%	87·6%	93·8%	83·0%
Received at least three other preventive health-care services* in the past year	52·2%	50·1%	9·5%	18·3%	49·3%	14·9%	74·7%	33·9%	42·4%	29·4%	33·8%	43·3%	51·8%	67·3%
Had unmet health-care needs in the past year	19·7%	19·9%	11·0%	6·1%	6·2%	21·4%	5·9%	16·6%	6·7%	25·8%	9·5%	22·4%	12·1%	18·8%
**Perceived quality and user experience**														
Rated quality of usual provider very good or excellent	61·0%	34·5%	36·8%	29·0%	45·5%	43·2%	43·8%	16·7%	44·5%	29·1%	56·4%	50·5%	53·7%	71·5%
Experienced discrimination in the health system	12·5%	6·9%	12·9%	3·8%	8·0%	10·7%	6·3%	12·0%	7·2%	14·3%	10·7%	6·6%	8·2%	6·8%
Believed medical mistake was made	12·3%	13·3%	6·0%	5·3%	9·2%	10·8%	8·5%	5·1%	5·7%	15·9%	8·9%	13·9%	10·4%	12·1%
**Health security**														
Confident could get and afford quality care	32·9%	30·7%	48·3%	69·2%	63·9%	43·0%	59·4%	71·3%	65·8%	26·4%	48·5%	48·8%	37·1%	57·7%
**Government responsiveness to public opinion**														
Confident government considers public input for health	27·2%	38·7%	79·6%	76·5%	40·8%	62·5%	52·9%	79·0%	73·7%	39·5%	51·6%	25·9%	37·0%	35·7%
**Government management of the COVID-19 pandemic**														
Rated government's management of pandemic as very good or excellent	39·0%	24·2%	53·9%	37·2%	25·0%	49·6%	30·8%	45·8%	25·6%	13·0%	39·4%	23·0%	54·9%	21·5%

Samples are representative of the adult population in each country except Argentina, where respondents represent the adult population of the Mendoza region only. All estimates include sampling weights. Survey questions for each health system quality measure are included in appendix 2 p 4.

* Had at least three of blood pressure check, blood cholesterol test, blood sugar test, dental examination, or eye examination.

## Associations between health-care use and perceived quality and COVID-19 vaccination

**Figure 2:**
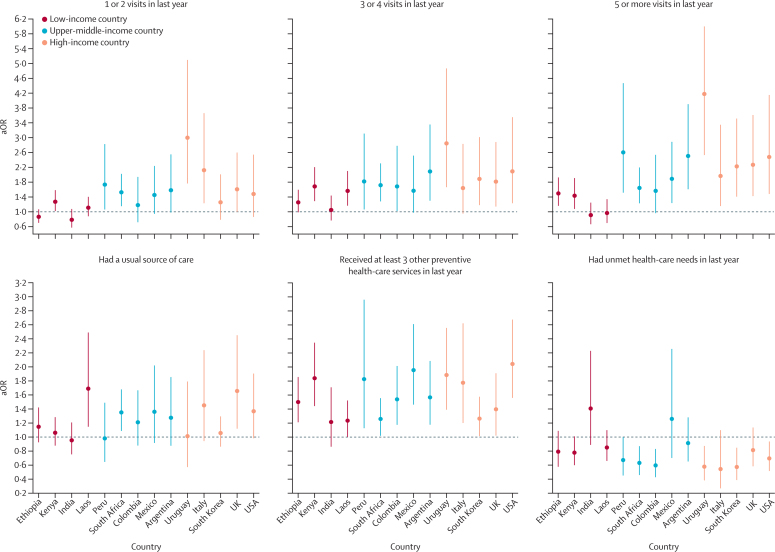
Associations between health-care use and health-system competence and COVID-19 vaccination Associations for respondents who had one or two, three or four, or five or more health-care visits in the past year; had a usual source of care; received at least three other preventive health-care services in the past year; or had unmet health-care needs in the past year. Countries are ordered according to their gross national income per capita (appendix 1 p 12). Full regression results are in appendix 2 (pp 8–15). aOR=adjusted odds ratio.

**Figure 3:**
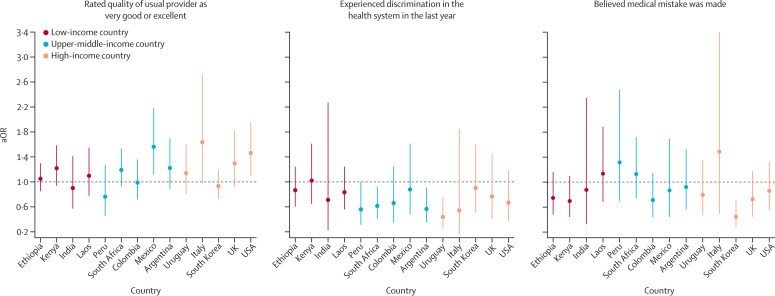
Associations between perceived quality and user experience and COVID-19 vaccination Associations for respondents who rated the quality of their usual provider as very good or excellent, experienced discrimination in the health system in the past year, or believed medical mistakes were made. Countries are ordered according to their gross national income per capita (appendix 1 p 12). aOR=adjusted odds ratio.

**Figure 4:**
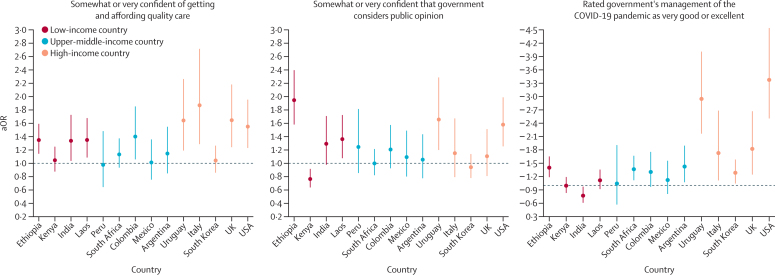
Health system confidence and COVID-19 vaccination Associations for respondents who were somewhat or very confident of getting and affording quality care if sick, somewhat or very confident that government considers public opinion, and who rated the government's management of the COVID-19 pandemic as very good or excellent. Countries are ordered according to their gross national income per capita (appendix 1 p 12). aOR=adjusted odds ratio.

Results from country-specific regression analyses are shown in Figure 2, Figure 3, Figure 4 and appendix 2 (pp 8–28). All variance inflation factor values were lower than 10, indicating only moderate to low correlation between the independent covariates. Results pooled across countries and by country-income group are presented in table 2. A greater number of health-care visits was associated with higher odds of COVID-19 vaccination in all countries except India (figure 2). Having a regular provider was positively associated with COVID-19 vaccination, but estimates were statistically different from the null only in Laos, South Africa, and the UK. Receiving at least three other preventive health services in the last year increased the odds of COVID-19 vaccination, with adjusted odds ratios (aORs) ranging from 1·23 (95% CI 1·00–1·52) for Laos to 2·04 (1·56–2·68) for the USA. By contrast, having unmet health-care needs reduced the odds of vaccination in approximately half the countries (figure 2).

**Table 2:** Health system use and quality and COVID-19 vaccination (at least two or three doses), pooled across countries and country-income groups

	**Low-income and lower-middle-income countries**	**Upper-middle-income countries**	**High-income countries**	**All countries**
**Health-care use in the past year**				
One to two visits	1·00 (0·81–1·24)	1·50 (1·25–1·79)	1·78 (1·32–2·40)	1·35 (1·13–1·61)
Three to four visits	1·37 (1·12–1·68)	1·75 (1·45–2·12)	2·00 (1·60–2·51)	1·63 (1·43–1·84)
Five or more visits	1·19 (0·92–1·53)	1·91 (1·57–2·31)	2·52 (1·96–3·24)	1·80 (1·46–2·21)
**Health system competence**				
Had a usual source of care	1·13 (0·95–1·35)	1·27 (1·10–1·46)	1·26 (1·04–1·52)	1·19 (1·09–1·31)
Received at least three other preventive health-care services in the past year	1·44 (1·19–1·74)	1·56 (1·32–1·84)	1·63 (1·33–1·99)	1·54 (1·39–1·70)
Had unmet health-care needs in the past year	0·88 (0·71–1·08)	0·74 (0·59–0·93)	0·67 (0·56–0·79)	0·75 (0·67–0·85)
**Perceived quality and user experience**				
Rated quality of usual provider as very good or excellent	1·09 (0·94–1·25)	1·16 (0·95–1·40)	1·22 (1·00–1·50)	1·15 (1·05–1·27)
Experienced discrimination in the health system	0·88 (0·71–1·11)	0·63 (0·50–0·80)	0·65 (0·49–0·86)	0·72 (0·63–0·83)
Believed medical mistake was made	0·82 (0·64–1·07)	0·96 (0·76–1·21)	0·72 (0·53–0·98)	0·83 (0·71–0·96)
**Health security**				
Confident could get and afford quality care	1·25 (1·10–1·43)	1·14 (1·01–1·29)	1·49 (1·19–1·87)	1·28 (1·16–1·41)
**Government responsiveness to public opinion**				
Believed government considers public input for health	1·27 (0·82–1·96)	1·09 (0·96–1·23)	1·25 (0·98–1·60)	1·20 (1·03–1·40)
**Government management of the COVID-19 pandemic**				
Rated government's management of pandemic as very good or excellent	1·06 (0·83–1·34)	1·31 (1·15–1·49)	2·09 (1·38–3·17)	1·42 (1·17–1·72)

Data are adjusted odds ratio (95% CI). Low-income and lower-middle-income countries are Ethiopia, India, Kenya, and Laos; upper-middle-income countries are Argentina, Colombia, Mexico, Peru, and South Africa; high-income countries are Italy, Korea, Uruguay, the UK, and the USA. Estimates from six distinct country-specific regression models were pooled across countries and country-income groups by inverse-variance-weighted random-effect meta-analysis.^15^ All underlying models were adjusted for age 50 years and older, chronic illness, past COVID-19, post-secondary education, residing in an urban area, female gender, and highest income group. In Ethiopia, Kenya, Laos, Mexico, Peru, South Africa, and the USA, models also included whether the respondent belonged to a minority ethnic, racial, or linguistic group.

Having a high-quality regular provider increased the odds of vaccination in Italy, Mexico, and the USA (figure 3). Experience of discrimination in the health system decreased the odds of COVID-19 vaccination in Argentina, South Africa, and Uruguay, whereas medical mistakes decreased the odds of vaccination in South Korea only (aOR 0·44, 95% CI 0·28–0·70). At least one confidence measure was statistically associated with COVID-19 vaccination in every country except Mexico and Peru. A sense of health security (being confident of accessing and affording quality care) increased the odds of COVID-19 vaccination in eight countries (aORs ranged from 1·34 [India] to 1·87 [Italy]; figure 4). Being confident that the government considers public opinion in making health decisions increased the odds of vaccination in Ethiopia, Laos, Uruguay, and the USA but decreased the odds of vaccination in Kenya (figure 4). Finally, supporting the government's management of the COVID-19 pandemic strongly increased the odds of vaccination in nine countries (aORs ranged from 1·29 [South Korea] to 3·37 [USA]) but slightly decreased the odds of vaccination in India (figure 4).

Being older than 50 years was generally associated with higher odds of vaccination (appendix 2 pp 8–28). Having a chronic illness increased the odds of COVID-19 vaccination in India and South Africa only. In Italy, Uruguay, and the USA, those who reported having COVID-19 had lower odds of vaccination. By contrast, in Mexico, Peru, and South Africa, previously having COVID-19 increased the odds of vaccination. Post-secondary education and higher income were generally associated with higher odds of COVID-19 vaccination. We found statistically significant differences in vaccination by gender in five countries. In India, South Korea, and the UK, women were less likely to be vaccinated, whereas in Kenya and South Africa, women were more likely to be vaccinated. Living in an urban area increased the odds of vaccination in Colombia, Italy, Laos, South Africa, and the USA but decreased the odds of vaccination in Ethiopia. Finally, lower odds of COVID-19 vaccination among minority ethnic or racial groups were found only in Laos and the UK, where those in the minority group were between 0·67-times and 0·56-times less likely to be vaccinated with three doses. By contrast, minority ethnic groups in Ethiopia had higher odds of vaccination (appendix 2 pp 8–28).

We found some patterns relating to country income and pandemic severity (table 2; appendix 2 p 30). Overall, the influence of health system use and quality on COVID-19 vaccination was stronger in high-income countries than in low-income or lower-middle-income countries (table 2). The estimates pooled across all 14 countries were statistically significant for all 12 predictors of interest. By contrast, several pooled estimates in the poorest four countries were not statistically different from the null. We also found a gradient in effect estimates according to the number of COVID-19-related deaths (appendix 2 p 30). The estimates were substantially higher in countries with the most COVID-19-related deaths, indicating that health systems might have stronger effects on vaccine decisions in countries significantly affected by the pandemic.

We also performed a sensitivity analysis using at least two doses of a COVID-19 vaccine as the outcome in all countries. We found that pooled estimates for the association between health system use and quality and COVID-19 vaccination with two or more doses were largely similar to the main analysis (appendix 2 p 29).

## Discussion

In this analysis, we used data from 14 countries to explore associations between a series of measures relating to health system use and quality and COVID-19 vaccination with at least two or at least three doses. We found that a greater number of health-care visits, having a regular provider, receiving other preventive health services, and having a high-quality regular provider were generally positively associated with COVID-19 vaccination. By contrast, having unmet health needs and experiencing discrimination or medical mistakes were negatively associated with COVID-19 vaccination. Confidence in the health system and the government also increased the odds of receiving at least two or three doses of a COVID-19 vaccine.

Our findings show that greater health-care use, better-quality health care, and having positive experiences in the health-care system were independently associated with COVID-19 vaccination, after controlling for several demographic and health-related factors, such as age, income, chronic illnesses, and education. These associations might, in part, result from the fact that people who have more frequent contact with the health system have more opportunities to receive a COVID-19 vaccine. However, decisions to be vaccinated also appear to be influenced by the quality of recent health-care experiences. Exposure to high-quality and respectful care can build confidence in the health system and in health-care interventions such as COVID-19 vaccination. Other studies have shown that health workers are the most trusted sources of guidance about COVID-19 vaccines and that they can act as trusted messengers in delivering information on health promotion.^7^, ^9^ Greater health-care use has also been linked to better uptake of human papillomavirus vaccination among teenage girls.^16^ Similarly, in our study, people who reported using at least three other preventive health services in the last year were considerably more likely to have had at least two or three doses of a COVID-19 vaccine. This finding might reflect individual attitudes, with people who are more proactive in taking care of their health being more likely to receive COVID-19 vaccines. Individuals with unmet health-care needs—ie, those who had forgone care despite needing medical attention—were less likely to have been vaccinated with at least two or three doses. These individuals might face greater barriers in accessing health care and could also be less engaged patients. Multiple studies have shown that people who score more highly on patient activation measures are more likely to engage in preventive behaviours such as immunisations.^17^ Strategies to improve patient engagement and activation—defined as the willingness and ability of individuals to take independent actions to manage their health and care—might also contribute to improved COVID-19 vaccination uptake.^17^

Having a regular source of care and perceiving the care received as being of high quality generally increased the odds of COVID-19 vaccination. Continuity of clinic and provider and having a regular primary care medical provider have been associated with increased uptake of health promotion such as preventive check-ups, cancer screenings, childhood immunisations, and influenza vaccinations.^18^, ^19^, ^20^, ^21^ In particular, the positive association between continuous primary care in the same clinic and vaccination in children is well documented.^18^, ^22^

Those who reported a medical mistake or discrimination were less likely to be vaccinated. Discrimination experienced within the health-care setting, or in society more generally, has been linked to negative patient experiences, lower levels of health-care-related trust, the delaying or forgoing of health care, and poorer treatment adherence.^23^

Consistent with other studies, we found that confidence in the health-care system and government increased the odds of COVID-19 vaccination.^24^ Others have shown higher levels of trust in government to be linked to a higher probability of COVID-19 vaccine acceptance.^24^ However, this association did not hold in Peru, which had the lowest levels of confidence in the health system and government but the highest rate of vaccination.

Among the demographic and health-related characteristics, we found that having previously had COVID-19 decreased the odds of vaccination in some high-income countries. Unfortunately, the temporal ordering of vaccination and previous COVID-19 was not known in our survey. Therefore, the negative association between illness and COVID-19 vaccination could simply result from the effectiveness of vaccines. It is also possible that despite the benefit of vaccination after COVID-19, some people might have decided that the SARS-CoV-2 infection provided enough immunity.^25^ By contrast, in middle-income countries, previous COVID-19 increased the odds of vaccination. In these countries, it is possible that having had COVID-19 motivated some individuals to seek vaccines; previous studies found that personal or family COVID-19 illness was positively associated with vaccine acceptance.^24^ Unlike in most other countries, urban respondents in Ethiopia were less likely to be vaccinated against COVID-19, compared with those living in rural areas. This difference might have resulted from strong outreach campaigns that took place in rural areas of Ethiopia where the health extension system is well developed.

Associations between health-care use and quality and COVID-19 vaccination were often stronger in high-income countries than in low-income or lower-middle-income countries. Vaccine decisions might be more strongly influenced by experiences in the health system in high-income countries, possibly reflecting a greater degree of patient activation and higher expectations in richer countries.^17^, ^26^ Estimates also tended to be higher in countries most affected by COVID-19-related deaths. This finding could indicate that past experiences and confidence in health systems might play a bigger role in changing vaccine decisions when countries are faced with a more severe public health crisis. By changing risk perceptions, differences in COVID-19 severity across countries might also directly affect people's willingness to get vaccinated.^4^

There are many other potential reasons why our findings varied across the 14 countries, including differences in health system structures, health policies, and insurance coverage. For example, despite having one of the highest rates of health-care resources per population, few people in South Korea have a primary care provider.^27^ The Korean National Health Insurance Service also covers most preventive health care, including dental and vision examinations.^27^ By contrast, the health systems of Ethiopia and Kenya are considerably underfunded, and regular access to preventive health care remains challenging. Across the 14 countries, health systems were predominantly public, government-owned, or based on social security, except in South Korea and the USA, where most health care is provided by the private sector.

Types of COVID-19 vaccine roll-out strategies, vaccine policies, and the types of institutions responsible for vaccination might also have affected our findings. For example, in Mexico, COVID-19 vaccination was organised by the army and national guard and provided solely in temporary mass-vaccination sites (outside of the health system) until April, 2022.^28^ Italy, India, Peru, and South Korea made COVID-19 vaccines mandatory for attending public venues and travelling.^29^ The influence of health system quality on COVID-19 vaccination in these contexts might, thus, be more limited. Timing of mass campaigns also varied; for example, the UK was one of the first countries to approve and begin administering COVID-19 vaccines in December, 2020, several months before some of the lower-income countries included in this Series paper.

Challenges in accessing COVID-19 vaccines in countries with low supplies probably also attenuated associations between health system quality and COVID-19 vaccination. In addition, the COVID-19 pandemic has affected health-care access and quality, as well as patient experiences globally, with these effects differing across countries.^30^ Finally, it is probable that variation in the underlying levels of COVID-19 vaccine hesitancy and misinformation across countries also affected our findings.^24^

Several studies have described the social and behavioural determinants of COVID-19 vaccine uptake.^1^, ^5^, ^6^, ^31^ Some have also shown a link between trust in government and COVID-19 vaccination.^24^ However, to our knowledge, no study has assessed the links between health system use and quality and COVID-19 vaccine uptake. Our study includes a large sample of respondents from various countries and contexts. Nonetheless, it has limitations. The validity of the self-reporting of numbers of COVID-19 doses received might be limited.^32^ The accuracy of our outcome measure identifying compliant or up-to-date vaccination is also limited by variation in national recommendations and the use of vaccine types requiring differing numbers of doses to complete a primary series. Several of the health-system measures we included related to health care received in the last year; however, it is possible that some (or all) of COVID-19 vaccine doses were received more than a year before the survey. Therefore, for this analysis, we assumed the reported health-care experience in the last year to be representative of health care received in recent years. There is also potential for reverse causality between the perceived quality of the health system or confidence in it and COVID-19 vaccination, whereby those who were able to access the COVID-19 vaccine quickly could be more likely to rate their health-care providers highly and to be satisfied with the government's management of the pandemic.

Our findings have implications for the development of strategies to promote vaccine uptake during health emergencies and highlight the central role that health-care providers play in promoting vaccination. A relationship of trust between patients and providers has been shown to reduce influenza vaccine hesitancy.^21^ Previous experimental studies have also shown that people prefer COVID-19 vaccines to be distributed by the health system rather than civil society groups or armed forces and that being able to get a vaccine at a primary care facility rather than a mass vaccination site could substantially reduce hesitancy.^9^, ^33^ Further supporting our findings, a recent study in the USA used causal inference methods to show that US counties with weak health-care systems (ie, fewer health-care providers per capita, lower preventive care, and lower health-care funding) had substantially lower COVID-19 vaccination coverage, adjusting for underlying rates of vaccine hesitancy, social vulnerability, and poverty.^34^

Recommended approaches to target the expansion of anti-vaccine activism have focused on public health messaging and on the coordinated distribution of vaccine-related information.^35^, ^36^ However, messaging alone might be insufficient to curb the rise in vaccine hesitancy. Ensuring that people can access affordable, respectful, and continuous care for all their health needs could help combat anti-vaccine attitudes and promote COVID-19 vaccination. Enabling such access includes increasing the number of people who have a regular provider or clinic, reducing unmet health-care needs, addressing discrimination in health care, and ensuring that people can build a relationship of trust with the health system. Building strong primary care systems and ensuring a baseline level of quality care that is affordable and accessible for all should be central to pandemic preparedness strategies.

## Data sharing

Individual-level, deidentified data from the People's Voice Survey will be publicly available in mid-2024. Data will be available on the Harvard Dataverse (https://dataverse.harvard.edu). The survey instrument and data dictionary will be available upon publication. The statistical code used for this analysis is available from an online repository (https://github.com/catherine-arsenault/PVS-HS-COVID-VACCINATION).

## Declaration of interests

Research for this Series paper was supported by grants to MEK and the QuEST Network from the Bill & Melinda Gates Foundation and the Swiss Federal Department of Foreign Affairs. This research was also supported by grants to CA and MEK from Merck Sharp & Dohme; to EGE and HHL from the Inter-American Development Bank; and to JO from the Taejae Foundation. The funders of the study had no role in study design or conduct, data collection, data management, data analysis, data interpretation, the writing of the Series paper, or the decision to submit the Series paper for publication.
