# Supplementary material for: Health system quality and COVID-19 vaccination: a cross-sectional analysis in 14 countries
Source: Lancet Glob Health. 2023 Dec 11;12(1):e156–65. doi: 10.1016/S2214-109X(23)00490-4 (PMC10716622; doi:10.1016/S2214-109X(23)00490-4)
Supplement: Supplementary appendix 2 [file mmc2.pdf]

THE LANCET
Global Health

Supplementary appendix 2

This appendix formed part of the original submission and has been peer reviewed. We post it as supplied by the authors.

Supplement to: Arsenault C, Lewis TP, Kapoor NR, et al. Health system quality and COVID-19 vaccination: a cross-sectional analysis in 14 countries. *Lancet Glob Health* 2023; published online Dec 11. https://doi.org/10.1016/S2214-109X(23)00490-4.

Health system quality and COVID-19 vaccination: a cross-sectional analysis in 14 countries

Appendix 2

SUPPLEMENTAL TABLE 1: SECURED DOSES OF COVID-19 VACCINES PER POPULATION IN EACH COUNTRY 2

SUPPLEMENTAL TABLE 2. SURVEY QUESTIONS TO ASSESS HEALTH SYSTEM UTILIZATION AND QUALITY 4

SUPPLEMENTAL TABLE 3. COUNTRY SPECIFIC DEFINITIONS OF MINORITY ETHNIC, RACIAL, OR LINGUISTIC GROUPS 6

SUPPLEMENTAL TABLE 4. CHARACTERISTICS OF RESPONDENTS BY COUNTRY 7

SUPPLEMENTAL TABLE 5. MODEL 1 HEALTH CARE UTILIZATION – COUNTRY-SPECIFIC REGRESSION RESULTS FOR THE ODDS OF COVID VACCINATION (2+ OR 3+ DOSES).. 8

SUPPLEMENTAL TABLE 6. MODEL 2 HEALTH SYSTEM COMPETENCE – COUNTRY-SPECIFIC REGRESSION RESULTS FOR THE ODDS OF COVID VACCINATION (2+ OR 3+ DOSES).. 12

SUPPLEMENTAL TABLE 7. MODEL 3 PERCEIVED QUALITY AND USER EXPERIENCE – COUNTRY-SPECIFIC REGRESSION RESULTS FOR THE ODDS OF COVID VACCINATION (2+ OR 3+ DOSES) 16

SUPPLEMENTAL TABLE 8. MODEL 4 HEALTH SECURITY – COUNTRY-SPECIFIC REGRESSION RESULTS FOR THE ODDS OF COVID VACCINATION (2+ OR 3+ DOSES) 20

SUPPLEMENTAL TABLE 9. MODEL 5 GOVERNMENT RESPONSIVENESS TO PUBLIC OPINION – COUNTRY-SPECIFIC REGRESSION RESULTS FOR THE ODDS OF COVID VACCINATION (2+ OR 3+ DOSES)... 23

SUPPLEMENTAL TABLE 10. MODEL 6 GOVERNMENT MANAGEMENT OF THE COVID-19 PANDEMIC – COUNTRY-SPECIFIC REGRESSION RESULTS FOR THE ODDS OF COVID VACCINATION (2+ OR 3+ DOSES)... 26

SUPPLEMENTAL TABLE 11. HEALTH SYSTEM UTILIZATION AND QUALITY AND COVID-19 VACCINATION WITH AT LEAST 2 DOSES, ADJUSTED ODDS RATIOS POOLED ACROSS COUNTRIES AND COUNTRY INCOME GROUPS 29

SUPPLEMENTAL TABLE 12. HEALTH SYSTEM UTILIZATION AND QUALITY AND COVID-19 VACCINATION (2+ OR 3+ DOSES), ADJUSTED ODDS RATIOS POOLED ACROSS COUNTRIES GROUPS BY COVID SEVERITY 30

Supplemental table 1: Secured doses of COVID-19 vaccines per population and comparison of vaccination rates in each country

Country	Income group [a]	Secured and/or Expected vaccine doses in percent of population [b]	Primary vaccination outcome included in the main analysis	Weighted proportion of survey respondents who received at least one dose of a COVID vaccine	Proportion of population who received at least one dose of a COVID vaccine at the time of the survey from administrative sources [c]
Ethiopia	Low	114	**2+ doses**	51.8%	35.0%
South Africa	Upper middle	117	**2+ doses**	60.8%	37.5%
Kenya	Lower middle	117	**2+ doses**	65.6%	25.0%
India	Lower middle	201	3+ doses	95.3%	72.5%
Mexico	Upper middle	247	3+ doses	90.8%	76.2%
Lao PDR	Lower middle	254	3+ doses	97.0%	77.3%
Colombia	Upper middle	274	3+ doses	90.2%	82.1%
Uruguay	High	275	3+ doses	90.7%	87.6%
Argentina	Upper middle	333	3+doses	94.2%	-
United States	High	397	3+ doses	76.9%	80.9%
South Korea	High	441	3+ doses	92.2%	86.4%
Peru	Upper middle	472	3+ doses	94.6%	87.5%
United Kingdom	High	669	3+ doses	95.5%	93.6% [d]
Italy	High	748	3+ doses	94.4%	86.2%

Countries are sorted by the number of secured and/or expected vaccine doses in percent of population.

a World Bank Country and Lending Groups, 2023
b IMF-WHO COVID-19 vaccine tracker, as of August 31, 2022: https://www.imf.org/en/Topics/imf-and-covid19/IMF-WHO-COVID-19-Vaccine-Tracker
c Source: Our World in Data Coronavirus (Covid-19) Vaccinations [accessed September 1, 2023]: https://ourworldindata.org/covid-vaccinations
d United Kingdom Office of National Statistics, Coronavirus (COVID-19) latest insights: Vaccines

Supplemental table 2. Survey questions to assess health system utilization and quality

Measure	Question	Response option
**Model 1: Health care utilization levels**		
Number of in-person health care visits in the last year	We are interested to hear about your use of health services in the past 12 months. Please answer only about your own experience. First, we will ask about only in-person visits, where you went to see a provider at a healthcare facility.<br><br>How many healthcare visits in total have you made in the past 12 months?	[Integer]
Number of home health care visits in the last year	Now we would like to ask you about home visits and virtual visits you had with healthcare providers.<br><br>How many visits did you have with a healthcare provider at your home in the past 12 months?	[Integer]
Number of telemedicine health care visits in the last year	How many virtual or telemedicine visits did you have in the past 12 months?<br><br>READ IF NECESSARY:<br>A telemedicine visit is when you use a phone or computer to have a visit with a healthcare provider rather than going to the healthcare facility in person. It does not include scheduling visits by phone or computer if you then go in person to the healthcare facility to meet with the provider.	[Integer]
**Model 2: Health system competence measures**		
Has a usual source of care	Is there one healthcare facility or healthcare provider's group you usually go to for most of your healthcare?	Yes/No
Received at least 3 other preventive health services	Please tell me, if you have received any of the following health services in the past 12 months from any healthcare provider:<br>- had your blood pressure tested<br>- had your eyes or vision checked<br>- had your teeth checked<br>- had a blood sugar test<br>- had a blood cholesterol test (this is checking for fat in the blood)	Yes/No
Had unmet health care needs in the past year	In the last twelve months, was there a time when you had a health problem and needed medical attention, but you did not get healthcare from a provider?	Yes/No
**Model 3: Perceived quality of care and user experience**		
Rates quality of usual provider very good or excellent	Overall, how would you rate the quality of healthcare you received in the past 12 months from this healthcare facility?	1 Excellent<br>2 Very good<br>3 Good<br>4 Fair<br>5 Poor

		6 I did not receive healthcare from this provider in the past 12 months
Experienced discrimination in the health system	Thinking about the last 12 months, have any of the following events happened to you personally? First, have you been treated unfairly or discriminated against by a doctor, nurse, or another healthcare provider?	Yes/No
Believes mistake was made in medical care	Thinking about the last 12 months, have any of the following events happened to you personally? Have you thought a medical mistake was made in your treatment or care? This could include a wrong diagnosis, incorrect type or amount of medication, or inappropriate sharing of private health information.	Yes/No
**Model 4: Health security**		
Confident can get and afford quality care	Now we would like to hear your thoughts on the healthcare system in your country as a whole, including public and private healthcare facilities and providers. How confident are you that:<br>- you would receive good quality healthcare if you became very sick<br>- you would be able to afford the healthcare you needed if you became very sick	1 Very confident<br>2 Somewhat confident<br>3 Not too confident<br>4 Not at all confident
**Model 5: Government responsiveness to public opinion**		
Confident government considers public input for health	How confident are you that the government considers the public's opinion when making decisions about the healthcare system?	1 Very confident<br>2 Somewhat confident<br>3 Not too confident<br>4 Not at all confident
**Model 6: Government's management of the COVID pandemic**		
Rates country management of COVID very good or excellent	How would you rate the government's management of the COVID-19 pandemic overall?	1 Excellent<br>2 Very good<br>3 Good<br>4 Fair<br>5 Poor

Supplemental table 3. Country specific definitions of minority ethnic, racial, or linguistic groups

Country	Minority group	Majority group
Ethiopia	Somaligna, Tigrigna, Sidamigna, Wolaytigna, Gurage, Afar, Hadiyya, Gamogna, Gedeo, Kafa	Amharegna, Oromiffa
Kenya	Embu, Kisii, Maasai, Meru, Mijikenda, Swahili, Somali, Taita/Taveta, Turkana, Samburu, Other	Kikuyu, Luhya, Kalenjin, Luo, Kamba
Lao PDR	Mon-Khmer (for q62a_la), Hmong-Lumien (for q62a_la), Other, Chinese-Tibetan	Lao-Tai
Mexico	Speaks an indigenous language	Does not speak an indigenous language
Peru	Quechua, Aimara, Ashaninka, Awajún/Aguaruna, Shipibo/Konibo, Shawi/Chayahuita, Matsigenka/ Machiguenga, Achua, other native language, other foreign language	Spanish
South Africa	Setswana, Xitsonga, isiNdebele, Tshivenda, siSwati	isiZulu, isiXhosa, Sesotho, Afrikaans, Sepedi, English
United Kingdom	Asian or Asian British, Black, Black British, Caribbean, or African, Mixed or multiple ethnic groups, Other ethnic group	White
United States	Hispanic or Latino, Black or African American, Asian, Native Hawaiian or other Pacific Islander, American Indian or Alaska Native (any combination of these responses)	White, Not hispanic or latino

Supplemental table 4. Characteristics of respondents by country

	Argentina N=1190	Colombia N=1237	Ethiopia N=2779	India N=2004	Italy N=1001	Kenya N=2305	Korea N=2000	Lao PDR N=2007	Mexico N=1002	Peru N=1255	South Africa N=2036	UK N=1677	Uruguay N=1237	USA N=1500
Age, mean (SD)	48.6 (16.0)	42.1 (16.3)	35.6 (15.2)	38.7 (14.9)	51.7 (17.5)	35.9 (14.3)	47.4 (15.0)	39.5 (14.3)	42.3 (16.3)	41.9 (16.0)	39.7 (15.1)	48.8 (18.3)	45.5 (16.8)	47.6 (17.7)
Has a chronic health problem	41.4%	27.1%	13.1%	14.6%	32.2%	15.7%	39.2%	23.9%	23.4%	24.9%	27.2%	50.9%	43.2%	40.7%
Had COVID-19	42.7%	28.4%	1.3%	7.0%	53.7%	6.1%	56.7%	15.8%	30.0%	45.9%	11.2%	69.7%	36.8%	54.8%
Completed post-secondary education	33.2%	21.5%	7.9%	37.5%	15.5%	10.7%	70.7%	15.2%	17.5%	17.0%	11.4%	75.5%	12.3%	61.6%
Highest income group	22.4%	33.8%	31.5%	14.2%	24.9%	30.7%	33.2%	49.5%	21.0%	15.4%	26.1%	37.6%	28.6%	41.1%
Female gender	61.3%	52.0%	49.9%	48.0%	52.0%	50.5%	50.4%	51.6%	52.9%	50.0%	51.3%	51.7%	52.3%	51.4%
Urban residence	93.9%	85.9%	29.7%	49.4%	95.2%	32.8%	86.8%	33.7%	78.0%	82.3%	68.8%	90.4%	92.5%	87.3%
Minority ethnic, racial, or linguistic group	-	-	27.9%	-	-	35.7%	-	31.7%	7.2%	14.3%	23.8%	10.1%	-	37.7%

Samples are representative of the adult population in each country except Argentina. In Argentina, sample represents the adult population of the Mendoza region only. All estimates include sampling weights.

Supplemental table 5. Model 1 health care utilization – Country-specific regression results for the odds of COVID vaccination (2+ or 3+ doses)

	aOR	p-value	LCL	UCL	Country	Income group
1-2 visits	1.61	0.052	1.00	2.60	UK	HI
3-4 visits	1.81	0.012	1.14	2.88	UK	HI
5 or more visits	2.27	0.001	1.42	3.61	UK	HI
Aged 50+	6.63	0.000	4.67	9.41	UK	HI
Has a chronic illness	1.18	0.266	0.88	1.59	UK	HI
Had COVID	0.93	0.657	0.69	1.26	UK	HI
Attended post-secondary education	1.85	0.000	1.33	2.56	UK	HI
Highest income group	1.90	0.000	1.40	2.56	UK	HI
Female	0.75	0.041	0.56	0.99	UK	HI
Urban	1.38	0.161	0.88	2.16	UK	HI
Minority ethnic or racial group	0.58	0.012	0.38	0.89	UK	HI
_cons	0.48	0.035	0.24	0.95	UK	HI
1-2 visits	1.48	0.154	0.86	2.54	USA	HI
3-4 visits	2.09	0.006	1.23	3.55	USA	HI
5 or more visits	2.48	0.001	1.48	4.15	USA	HI
Aged 50+	2.33	0.000	1.84	2.96	USA	HI
Has a chronic illness	1.12	0.364	0.88	1.42	USA	HI
Had COVID	0.69	0.001	0.55	0.87	USA	HI
Attended post-secondary education	2.42	0.000	1.88	3.12	USA	HI
Highest income group	1.69	0.000	1.33	2.15	USA	HI
Female	0.91	0.413	0.72	1.14	USA	HI
Urban	1.66	0.006	1.15	2.38	USA	HI
Minority ethnic or racial group	1.14	0.285	0.90	1.45	USA	HI
_cons	0.10	0.000	0.05	0.19	USA	HI
1-2 visits	1.53	0.003	1.15	2.02	South Africa	UMI
3-4 visits	1.72	0.000	1.28	2.31	South Africa	UMI
5 or more visits	1.64	0.001	1.23	2.20	South Africa	UMI
Aged 50+	2.24	0.000	1.67	3.01	South Africa	UMI
Has a chronic illness	1.67	0.000	1.31	2.14	South Africa	UMI
Had COVID	1.79	0.000	1.35	2.37	South Africa	UMI
Attended post-secondary education	1.23	0.058	0.99	1.53	South Africa	UMI
Highest income group	1.31	0.013	1.06	1.61	South Africa	UMI
Female	1.33	0.004	1.09	1.62	South Africa	UMI
Urban	1.57	0.000	1.24	1.98	South Africa	UMI
Minority ethnic or racial group	0.93	0.493	0.74	1.15	South Africa	UMI
_cons	0.17	0.000	0.12	0.23	South Africa	UMI
1-2 visits	1.73	0.027	1.06	2.83	Peru	UMI
3-4 visits	1.82	0.029	1.06	3.11	Peru	UMI
5 or more visits	2.60	0.001	1.52	4.47	Peru	UMI
Aged 50+	1.45	0.147	0.88	2.40	Peru	UMI
Has a chronic illness	0.78	0.324	0.48	1.28	Peru	UMI
Had COVID	1.87	0.002	1.27	2.75	Peru	UMI
Attended post-secondary education	2.66	0.000	1.68	4.20	Peru	UMI
Highest income group	1.84	0.046	1.01	3.34	Peru	UMI
Female	1.19	0.345	0.83	1.72	Peru	UMI
Urban	0.99	0.983	0.55	1.79	Peru	UMI
Minority ethnic or racial group	0.70	0.204	0.40	1.21	Peru	UMI
_cons	2.19	0.028	1.09	4.38	Peru	UMI
1-2 visits	1.45	0.089	0.94	2.24	Mexico	UMI
3-4 visits	1.57	0.062	0.98	2.51	Mexico	UMI
5 or more visits	1.89	0.003	1.24	2.89	Mexico	UMI

	aOR	p-value	LCL	UCL	Country	Income group
Aged 50+	2.43	0.000	1.76	3.35	Mexico	UMI
Has a chronic illness	1.08	0.670	0.76	1.55	Mexico	UMI
Had COVID	1.33	0.060	0.99	1.79	Mexico	UMI
Attended post-secondary education	1.36	0.066	0.98	1.90	Mexico	UMI
Highest income group	1.02	0.910	0.72	1.45	Mexico	UMI
Female	1.02	0.895	0.76	1.38	Mexico	UMI
Urban	1.15	0.451	0.80	1.65	Mexico	UMI
Minority ethnic or racial group	0.74	0.266	0.43	1.26	Mexico	UMI
_cons	0.50	0.006	0.31	0.82	Mexico	UMI
1-2 visits	1.11	0.375	0.88	1.40	Lao PDR	LMI
3-4 visits	1.56	0.003	1.16	2.10	Lao PDR	LMI
5 or more visits	0.97	0.845	0.70	1.34	Lao PDR	LMI
Aged 50+	1.51	0.000	1.20	1.89	Lao PDR	LMI
Has a chronic illness	0.70	0.003	0.56	0.89	Lao PDR	LMI
Had COVID	0.99	0.929	0.78	1.26	Lao PDR	LMI
Attended post-secondary education	1.73	0.000	1.40	2.13	Lao PDR	LMI
Highest income group	1.29	0.016	1.05	1.59	Lao PDR	LMI
Female	1.15	0.175	0.94	1.40	Lao PDR	LMI
Urban	1.33	0.009	1.07	1.64	Lao PDR	LMI
Minority ethnic or racial group	0.63	0.000	0.50	0.80	Lao PDR	LMI
_cons	0.69	0.006	0.53	0.90	Lao PDR	LMI
1-2 visits	1.27	0.032	1.02	1.58	Kenya	LMI
3-4 visits	1.68	0.000	1.29	2.20	Kenya	LMI
5 or more visits	1.43	0.013	1.08	1.91	Kenya	LMI
Aged 50+	2.07	0.000	1.53	2.79	Kenya	LMI
Has a chronic illness	1.09	0.584	0.81	1.46	Kenya	LMI
Had COVID	1.18	0.445	0.77	1.83	Kenya	LMI
Attended post-secondary education	1.60	0.000	1.31	1.96	Kenya	LMI
Highest income group	1.33	0.004	1.10	1.62	Kenya	LMI
Female	1.20	0.053	1.00	1.45	Kenya	LMI
Urban	1.00	0.979	0.82	1.22	Kenya	LMI
Minority ethnic or racial group	0.89	0.267	0.73	1.09	Kenya	LMI
_cons	0.52	0.000	0.41	0.65	Kenya	LMI
1-2 visits	2.12	0.007	1.23	3.66	Italy	HI
3-4 visits	1.64	0.075	0.95	2.83	Italy	HI
5 or more visits	1.97	0.013	1.16	3.35	Italy	HI
Aged 50+	2.94	0.000	2.01	4.31	Italy	HI
Has a chronic illness	1.13	0.566	0.74	1.72	Italy	HI
Had COVID	0.34	0.000	0.23	0.51	Italy	HI
Attended post-secondary education	1.16	0.477	0.77	1.74	Italy	HI
Highest income group	1.46	0.083	0.95	2.26	Italy	HI
Female	0.91	0.609	0.62	1.32	Italy	HI
Urban	1.81	0.203	0.73	4.49	Italy	HI
_cons	1.51	0.426	0.55	4.19	Italy	HI
_cons	1.29	0.633	0.46	3.64	Italy	HI
1-2 visits	3.00	0.000	1.76	5.10	Uruguay	HI
3-4 visits	2.85	0.000	1.66	4.87	Uruguay	HI
5 or more visits	4.18	0.000	2.53	6.89	Uruguay	HI
Aged 50+	2.17	0.000	1.54	3.04	Uruguay	HI
Has a chronic illness	1.25	0.210	0.88	1.77	Uruguay	HI
Had COVID	0.68	0.013	0.51	0.92	Uruguay	HI
Attended post-secondary education	1.66	0.008	1.14	2.43	Uruguay	HI
Highest income group	1.89	0.001	1.31	2.71	Uruguay	HI
Female	1.05	0.765	0.77	1.43	Uruguay	HI
Urban	0.65	0.188	0.34	1.24	Uruguay	HI

	aOR	p-value	LCL	UCL	Country	Income group
_cons	1.01	0.971	0.47	2.19	Uruguay	HI
_cons	1.15	0.765	0.47	2.81	Uruguay	HI
1-2 visits	1.26	0.339	0.79	2.01	Korea	HI
3-4 visits	1.89	0.008	1.18	3.01	Korea	HI
5 or more visits	2.23	0.001	1.41	3.51	Korea	HI
Aged 50+	2.91	0.000	2.38	3.54	Korea	HI
Has a chronic illness	1.02	0.857	0.83	1.26	Korea	HI
Had COVID	0.85	0.099	0.69	1.03	Korea	HI
Attended post-secondary education	1.12	0.277	0.91	1.39	Korea	HI
Highest income group	1.06	0.562	0.86	1.31	Korea	HI
Female	0.77	0.007	0.64	0.93	Korea	HI
Urban	1.06	0.688	0.80	1.40	Korea	HI
_cons	0.60	0.053	0.36	1.01	Korea	HI
_cons	0.61	0.057	0.36	1.02	Korea	HI
1-2 visits	0.79	0.133	0.57	1.08	India	LMI
3-4 visits	1.05	0.761	0.77	1.44	India	LMI
5 or more visits	0.91	0.564	0.67	1.25	India	LMI
Aged 50+	1.51	0.059	0.98	2.31	India	LMI
Has a chronic illness	1.42	0.084	0.95	2.10	India	LMI
Had COVID	1.01	0.985	0.58	1.75	India	LMI
Attended post-secondary education	0.95	0.650	0.75	1.20	India	LMI
Highest income group	1.58	0.007	1.14	2.20	India	LMI
Female	0.81	0.080	0.64	1.03	India	LMI
Urban	0.99	0.936	0.79	1.25	India	LMI
_cons	0.38	0.000	0.29	0.49	India	LMI
_cons	0.39	0.000	0.30	0.51	India	LMI
1-2 visits	1.18	0.511	0.72	1.94	Colombia	UMI
3-4 visits	1.68	0.041	1.02	2.78	Colombia	UMI
5 or more visits	1.56	0.069	0.97	2.54	Colombia	UMI
Aged 50+	4.38	0.000	3.25	5.91	Colombia	UMI
Has a chronic illness	1.07	0.686	0.78	1.46	Colombia	UMI
Had COVID	0.95	0.703	0.72	1.25	Colombia	UMI
Attended post-secondary education	1.64	0.001	1.22	2.21	Colombia	UMI
Highest income group	1.44	0.012	1.08	1.91	Colombia	UMI
Female	1.17	0.262	0.89	1.53	Colombia	UMI
Urban	2.01	0.002	1.29	3.14	Colombia	UMI
_cons	0.09	0.000	0.05	0.16	Colombia	UMI
_cons	0.08	0.000	0.05	0.16	Colombia	UMI
1-2 visits	1.58	0.059	0.98	2.55	Argentina	UMI
3-4 visits	2.09	0.002	1.30	3.35	Argentina	UMI
5 or more visits	2.51	0.000	1.61	3.90	Argentina	UMI
Aged 50+	3.14	0.000	2.33	4.24	Argentina	UMI
Has a chronic illness	1.15	0.351	0.85	1.56	Argentina	UMI
Had COVID	0.94	0.635	0.71	1.23	Argentina	UMI
Attended post-secondary education	1.39	0.036	1.02	1.90	Argentina	UMI
Highest income group	1.36	0.098	0.94	1.95	Argentina	UMI
Female	1.10	0.513	0.83	1.47	Argentina	UMI
Urban	0.96	0.898	0.54	1.72	Argentina	UMI
_cons	0.57	0.094	0.29	1.10	Argentina	UMI
_cons	0.57	0.112	0.28	1.14	Argentina	UMI
1-2 visits	0.87	0.180	0.70	1.07	Ethiopia	LMI
3-4 visits	1.25	0.063	0.99	1.59	Ethiopia	LMI
5 or more visits	1.50	0.002	1.16	1.93	Ethiopia	LMI
Aged 50+	1.11	0.385	0.88	1.41	Ethiopia	LMI
Has a chronic illness	1.12	0.355	0.89	1.41	Ethiopia	LMI

	aOR	p-value	LCL	UCL	Country	Income group
Had COVID	1.18	0.544	0.69	2.01	Ethiopia	LMI
Attended post-secondary education	1.18	0.093	0.97	1.43	Ethiopia	LMI
Highest income group	0.87	0.152	0.73	1.05	Ethiopia	LMI
Female	1.02	0.862	0.86	1.20	Ethiopia	LMI
Urban	0.69	0.000	0.57	0.84	Ethiopia	LMI
Minority ethnic or racial group	1.81	0.000	1.50	2.19	Ethiopia	LMI
_cons	0.56	0.000	0.45	0.70	Ethiopia	LMI

aOR is adjusted Odds Ratio. LCL and UCL are lower- and upper confidence limits of the 95% confidence interval. LMI are low- and lower-middle-income countries. UMI are upper-middle-income countries. HI are high-income countries. _cons represents the baseline odds. Regressions are performed separately in each country.

Supplemental table 6. Model 2 Health system competence – Country-specific regression results for the odds of COVID vaccination (2+ or 3+ doses)

	aOR	p-value	LCL	UCL	Country	Income group
Has a usual health facility or provider	1.66	0.012	1.12	2.45	UK	HI
Received at least 3 other preventive health services	1.40	0.036	1.02	1.91	UK	HI
Had unmet health care need in last year	0.81	0.225	0.58	1.14	UK	HI
Aged 50+	5.94	0.000	4.10	8.62	UK	HI
Has a chronic illness	1.28	0.099	0.95	1.70	UK	HI
Had COVID	0.89	0.478	0.65	1.22	UK	HI
Attended post-secondary education	1.79	0.001	1.28	2.50	UK	HI
Highest income group	1.86	0.000	1.38	2.52	UK	HI
Female	0.77	0.065	0.58	1.02	UK	HI
Urban	1.44	0.118	0.91	2.28	UK	HI
Minority ethnic or racial group	0.60	0.022	0.39	0.93	UK	HI
_cons	0.53	0.068	0.26	1.05	UK	HI
Has a usual health facility or provider	1.37	0.063	0.98	1.90	USA	HI
Received at least 3 other preventive health services	2.04	0.000	1.56	2.68	USA	HI
Had unmet health care need in last year	0.70	0.017	0.52	0.94	USA	HI
Aged 50+	1.91	0.000	1.48	2.45	USA	HI
Has a chronic illness	1.23	0.086	0.97	1.56	USA	HI
Had COVID	0.71	0.003	0.56	0.89	USA	HI
Attended post-secondary education	2.42	0.000	1.87	3.12	USA	HI
Highest income group	1.51	0.001	1.18	1.93	USA	HI
Female	0.97	0.778	0.77	1.22	USA	HI
Urban	1.74	0.003	1.21	2.49	USA	HI
Minority ethnic or racial group	1.14	0.287	0.89	1.46	USA	HI
_cons	0.10	0.000	0.06	0.18	USA	HI
Has a usual health facility or provider	1.35	0.007	1.09	1.68	South Africa	UMI
Received at least 3 other preventive health services	1.26	0.033	1.02	1.56	South Africa	UMI
Had unmet health care need in last year	0.63	0.005	0.46	0.87	South Africa	UMI
Aged 50+	2.08	0.000	1.55	2.81	South Africa	UMI
Has a chronic illness	1.72	0.000	1.35	2.20	South Africa	UMI
Had COVID	1.77	0.000	1.33	2.35	South Africa	UMI
Attended post-secondary education	1.19	0.114	0.96	1.47	South Africa	UMI
Highest income group	1.23	0.050	1.00	1.52	South Africa	UMI
Female	1.37	0.002	1.13	1.66	South Africa	UMI
Urban	1.54	0.000	1.21	1.95	South Africa	UMI
Minority ethnic or racial group	0.96	0.729	0.77	1.20	South Africa	UMI
_cons	0.20	0.000	0.15	0.27	South Africa	UMI
Has a usual health facility or provider	0.98	0.929	0.65	1.49	Peru	UMI
Received at least 3 other preventive health services	1.83	0.014	1.13	2.96	Peru	UMI
Had unmet health care need in last year	0.67	0.050	0.45	1.00	Peru	UMI
Aged 50+	1.33	0.273	0.80	2.23	Peru	UMI
Has a chronic illness	0.98	0.919	0.61	1.57	Peru	UMI
Had COVID	1.98	0.001	1.35	2.92	Peru	UMI
Attended post-secondary education	2.68	0.000	1.70	4.22	Peru	UMI
Highest income group	1.71	0.083	0.93	3.12	Peru	UMI
Female	1.30	0.157	0.90	1.86	Peru	UMI
Urban	0.96	0.897	0.54	1.72	Peru	UMI
Minority ethnic or racial group	0.72	0.240	0.42	1.25	Peru	UMI
_cons	3.54	0.000	1.77	7.07	Peru	UMI
Has a usual health facility or provider	1.36	0.127	0.92	2.02	Mexico	UMI
Received at least 3 other preventive health services	1.96	0.000	1.46	2.61	Mexico	UMI
Had unmet health care need in last year	1.26	0.440	0.70	2.26	Mexico	UMI
Aged 50+	2.31	0.000	1.67	3.18	Mexico	UMI

	aOR	p-value	LCL	UCL	Country	Income group
Has a chronic illness	0.95	0.774	0.66	1.36	Mexico	UMI
Had COVID	1.31	0.076	0.97	1.77	Mexico	UMI
Attended post-secondary education	1.30	0.126	0.93	1.82	Mexico	UMI
Highest income group	1.01	0.961	0.71	1.44	Mexico	UMI
Female	1.05	0.752	0.78	1.42	Mexico	UMI
Urban	1.13	0.495	0.79	1.62	Mexico	UMI
Minority ethnic or racial group	0.74	0.272	0.43	1.27	Mexico	UMI
_cons	0.45	0.003	0.27	0.76	Mexico	UMI
Has a usual health facility or provider	1.69	0.008	1.15	2.49	Lao PDR	LMI
Received at least 3 other preventive health services	1.23	0.048	1.00	1.52	Lao PDR	LMI
Had unmet health care need in last year	0.85	0.214	0.66	1.10	Lao PDR	LMI
Aged 50+	1.45	0.002	1.15	1.83	Lao PDR	LMI
Has a chronic illness	0.68	0.001	0.54	0.86	Lao PDR	LMI
Had COVID	0.97	0.829	0.76	1.24	Lao PDR	LMI
Attended post-secondary education	1.70	0.000	1.38	2.10	Lao PDR	LMI
Highest income group	1.27	0.025	1.03	1.57	Lao PDR	LMI
Female	1.11	0.296	0.91	1.36	Lao PDR	LMI
Urban	1.36	0.005	1.10	1.69	Lao PDR	LMI
Minority ethnic or racial group	0.67	0.001	0.53	0.85	Lao PDR	LMI
_cons	0.46	0.000	0.29	0.71	Lao PDR	LMI
Has a usual health facility or provider	1.06	0.540	0.88	1.28	Kenya	LMI
Received at least 3 other preventive health services	1.84	0.000	1.44	2.35	Kenya	LMI
Had unmet health care need in last year	0.78	0.059	0.60	1.01	Kenya	LMI
Aged 50+	1.88	0.000	1.38	2.56	Kenya	LMI
Has a chronic illness	1.18	0.269	0.88	1.60	Kenya	LMI
Had COVID	1.18	0.477	0.75	1.84	Kenya	LMI
Attended post-secondary education	1.59	0.000	1.30	1.95	Kenya	LMI
Highest income group	1.29	0.012	1.06	1.57	Kenya	LMI
Female	1.24	0.023	1.03	1.49	Kenya	LMI
Urban	0.96	0.647	0.78	1.16	Kenya	LMI
Minority ethnic or racial group	0.91	0.341	0.74	1.11	Kenya	LMI
_cons	0.61	0.000	0.49	0.76	Kenya	LMI
Has a usual health facility or provider	1.45	0.091	0.94	2.24	Italy	HI
Received at least 3 other preventive health services	1.78	0.004	1.20	2.62	Italy	HI
Had unmet health care need in last year	0.54	0.089	0.27	1.10	Italy	HI
Aged 50+	2.50	0.000	1.67	3.73	Italy	HI
Has a chronic illness	1.13	0.570	0.74	1.74	Italy	HI
Had COVID	0.33	0.000	0.22	0.51	Italy	HI
Attended post-secondary education	1.20	0.394	0.79	1.82	Italy	HI
Highest income group	1.40	0.136	0.90	2.17	Italy	HI
Female	0.92	0.686	0.63	1.35	Italy	HI
Urban	1.75	0.202	0.74	4.12	Italy	HI
_cons	1.71	0.267	0.66	4.40	Italy	HI
Has a usual health facility or provider	1.01	0.966	0.57	1.79	Uruguay	HI
Received at least 3 other preventive health services	1.89	0.000	1.39	2.56	Uruguay	HI
Had unmet health care need in last year	0.58	0.009	0.38	0.87	Uruguay	HI
Aged 50+	1.91	0.000	1.37	2.68	Uruguay	HI
Has a chronic illness	1.42	0.037	1.02	1.97	Uruguay	HI
Had COVID	0.80	0.132	0.59	1.07	Uruguay	HI
Attended post-secondary education	1.64	0.009	1.13	2.38	Uruguay	HI
Highest income group	1.86	0.001	1.31	2.65	Uruguay	HI
Female	1.18	0.280	0.87	1.59	Uruguay	HI
Urban	0.70	0.309	0.36	1.39	Uruguay	HI
_cons	2.00	0.115	0.84	4.76	Uruguay	HI
Has a usual health facility or provider	1.06	0.590	0.86	1.29	Korea	HI

	aOR	p-value	LCL	UCL	Country	Income group
Received at least 3 other preventive health services	1.26	0.037	1.01	1.58	Korea	HI
Had unmet health care need in last year	0.57	0.005	0.39	0.85	Korea	HI
Aged 50+	2.77	0.000	2.27	3.38	Korea	HI
Has a chronic illness	1.18	0.110	0.96	1.45	Korea	HI
Had COVID	0.93	0.453	0.76	1.13	Korea	HI
Attended post-secondary education	1.12	0.308	0.90	1.38	Korea	HI
Highest income group	1.05	0.646	0.85	1.29	Korea	HI
Female	0.80	0.023	0.66	0.97	Korea	HI
Urban	1.06	0.694	0.80	1.40	Korea	HI
_cons	0.82	0.295	0.56	1.19	Korea	HI
Has a usual health facility or provider	0.95	0.694	0.75	1.21	India	LMI
Received at least 3 other preventive health services	1.22	0.264	0.86	1.71	India	LMI
Had unmet health care need in last year	1.41	0.146	0.89	2.23	India	LMI
Aged 50+	1.48	0.073	0.96	2.27	India	LMI
Has a chronic illness	1.38	0.106	0.93	2.03	India	LMI
Had COVID	1.00	0.991	0.58	1.74	India	LMI
Attended post-secondary education	0.94	0.581	0.74	1.19	India	LMI
Highest income group	1.58	0.007	1.14	2.20	India	LMI
Female	0.82	0.093	0.64	1.03	India	LMI
Urban	0.98	0.875	0.78	1.24	India	LMI
_cons	0.35	0.000	0.28	0.45	India	LMI
Has a usual health facility or provider	1.21	0.241	0.88	1.67	Colombia	UMI
Received at least 3 other preventive health services	1.54	0.002	1.18	2.01	Colombia	UMI
Had unmet health care need in last year	0.60	0.002	0.43	0.83	Colombia	UMI
Aged 50+	4.08	0.000	3.01	5.54	Colombia	UMI
Has a chronic illness	1.11	0.529	0.81	1.52	Colombia	UMI
Had COVID	0.97	0.852	0.73	1.29	Colombia	UMI
Attended post-secondary education	1.63	0.001	1.21	2.20	Colombia	UMI
Highest income group	1.37	0.030	1.03	1.82	Colombia	UMI
Female	1.16	0.263	0.89	1.52	Colombia	UMI
Urban	2.00	0.002	1.29	3.11	Colombia	UMI
_cons	0.09	0.000	0.05	0.16	Colombia	UMI
Has a usual health facility or provider	1.28	0.204	0.88	1.85	Argentina	UMI
Received at least 3 other preventive health services	1.57	0.002	1.18	2.09	Argentina	UMI
Had unmet health care need in last year	0.91	0.601	0.65	1.28	Argentina	UMI
Aged 50+	2.85	0.000	2.11	3.85	Argentina	UMI
Has a chronic illness	1.20	0.247	0.88	1.62	Argentina	UMI
Had COVID	0.95	0.707	0.72	1.25	Argentina	UMI
Attended post-secondary education	1.37	0.045	1.01	1.87	Argentina	UMI
Highest income group	1.33	0.121	0.93	1.91	Argentina	UMI
Female	1.16	0.288	0.88	1.54	Argentina	UMI
Urban	1.02	0.935	0.57	1.85	Argentina	UMI
_cons	0.69	0.281	0.35	1.36	Argentina	UMI
Has a usual health facility or provider	1.15	0.211	0.93	1.42	Ethiopia	LMI
Received at least 3 other preventive health services	1.50	0.000	1.21	1.86	Ethiopia	LMI
Had unmet health care need in last year	0.79	0.150	0.58	1.09	Ethiopia	LMI
Aged 50+	1.11	0.405	0.87	1.40	Ethiopia	LMI
Has a chronic illness	1.19	0.136	0.95	1.49	Ethiopia	LMI
Had COVID	1.10	0.737	0.64	1.87	Ethiopia	LMI
Attended post-secondary education	1.17	0.120	0.96	1.42	Ethiopia	LMI
Highest income group	0.86	0.109	0.71	1.03	Ethiopia	LMI
Female	1.01	0.882	0.85	1.20	Ethiopia	LMI
Urban	0.68	0.000	0.56	0.83	Ethiopia	LMI
Minority ethnic or racial group	1.88	0.000	1.56	2.27	Ethiopia	LMI
_cons	0.51	0.000	0.39	0.67	Ethiopia	LMI

aOR is adjusted Odds Ratio. LCL and UCL are lower- and upper confidence limits of the 95% confidence interval. LMI are low- and lower-middle-income countries. UMI are upper-middle-income countries. HI are high-income countries. _cons represents the baseline odds. Regressions are performed separately in each country.

Supplemental table 7. Model 3 perceived quality and user experience – Country-specific regression results for the odds of COVID vaccination (2+ or 3+ doses)

	aOR	p-value	LCL	UCL	Country	Income group
Rates usual facility/provider quality as very good or excellent	1.30	0.133	0.92	1.82	UK	HI
Experienced discrimination in health system	0.77	0.418	0.40	1.46	UK	HI
Believes medical error was made in care	0.73	0.192	0.45	1.17	UK	HI
Aged 50+	7.55	0.000	4.92	11.59	UK	HI
Has a chronic illness	1.33	0.098	0.95	1.85	UK	HI
Had COVID	0.90	0.590	0.61	1.33	UK	HI
Attended post-secondary education	1.55	0.036	1.03	2.33	UK	HI
Highest income group	1.97	0.000	1.36	2.85	UK	HI
Female	0.66	0.018	0.46	0.93	UK	HI
Urban	1.22	0.460	0.72	2.09	UK	HI
Minority ethnic or racial group	0.58	0.044	0.34	0.99	UK	HI
_cons	1.23	0.602	0.57	2.64	UK	HI
Rates usual facility/provider quality as very good or excellent	1.46	0.011	1.09	1.96	USA	HI
Experienced discrimination in health system	0.67	0.168	0.37	1.19	USA	HI
Believes medical error was made in care	0.86	0.495	0.56	1.32	USA	HI
Aged 50+	2.26	0.000	1.73	2.96	USA	HI
Has a chronic illness	1.23	0.112	0.95	1.59	USA	HI
Had COVID	0.60	0.000	0.47	0.78	USA	HI
Attended post-secondary education	2.53	0.000	1.91	3.35	USA	HI
Highest income group	1.35	0.029	1.03	1.76	USA	HI
Female	0.94	0.624	0.72	1.22	USA	HI
Urban	1.64	0.012	1.11	2.43	USA	HI
Minority ethnic or racial group	1.13	0.374	0.86	1.48	USA	HI
_cons	0.21	0.000	0.12	0.38	USA	HI
Rates usual facility/provider quality as very good or excellent	1.19	0.175	0.93	1.53	South Africa	UMI
Experienced discrimination in health system	0.62	0.020	0.41	0.93	South Africa	UMI
Believes medical error was made in care	1.13	0.572	0.74	1.72	South Africa	UMI
Aged 50+	2.08	0.000	1.44	3.01	South Africa	UMI
Has a chronic illness	1.60	0.001	1.21	2.13	South Africa	UMI
Had COVID	1.55	0.013	1.10	2.20	South Africa	UMI
Attended post-secondary education	1.24	0.127	0.94	1.62	South Africa	UMI
Highest income group	1.39	0.018	1.06	1.82	South Africa	UMI
Female	1.42	0.006	1.10	1.81	South Africa	UMI
Urban	1.36	0.046	1.01	1.84	South Africa	UMI
Minority ethnic or racial group	0.80	0.140	0.60	1.08	South Africa	UMI
_cons	0.31	0.000	0.22	0.45	South Africa	UMI
Rates usual facility/provider quality as very good or excellent	0.76	0.300	0.46	1.27	Peru	UMI
Experienced discrimination in health system	0.56	0.050	0.31	1.00	Peru	UMI
Believes medical error was made in care	1.32	0.395	0.70	2.48	Peru	UMI
Aged 50+	0.98	0.952	0.53	1.82	Peru	UMI
Has a chronic illness	0.89	0.707	0.49	1.62	Peru	UMI
Had COVID	1.89	0.010	1.17	3.07	Peru	UMI
Attended post-secondary education	2.04	0.013	1.16	3.59	Peru	UMI
Highest income group	2.02	0.063	0.96	4.24	Peru	UMI
Female	1.19	0.459	0.75	1.90	Peru	UMI
Urban	1.16	0.700	0.54	2.47	Peru	UMI
Minority ethnic or racial group	0.75	0.423	0.38	1.51	Peru	UMI
_cons	4.48	0.001	1.85	10.89	Peru	UMI
Rates usual facility/provider quality as very good or excellent	1.56	0.009	1.12	2.18	Mexico	UMI
Experienced discrimination in health system	0.88	0.676	0.48	1.61	Mexico	UMI

	aOR	p-value	LCL	UCL	Country	Income group
Believes medical error was made in care	0.87	0.675	0.44	1.69	Mexico	UMI
Aged 50+	2.78	0.000	1.87	4.13	Mexico	UMI
Has a chronic illness	1.06	0.787	0.70	1.60	Mexico	UMI
Had COVID	1.71	0.003	1.21	2.43	Mexico	UMI
Attended post-secondary education	1.20	0.342	0.82	1.77	Mexico	UMI
Highest income group	1.16	0.493	0.76	1.77	Mexico	UMI
Female	1.22	0.290	0.84	1.77	Mexico	UMI
Urban	1.16	0.500	0.75	1.80	Mexico	UMI
Minority ethnic or racial group	0.70	0.244	0.38	1.28	Mexico	UMI
_cons	0.54	0.020	0.32	0.91	Mexico	UMI
Rates usual facility/provider quality as very good or excellent	1.10	0.599	0.78	1.55	Lao PDR	LMI
Experienced discrimination in health system	0.83	0.372	0.56	1.24	Lao PDR	LMI
Believes medical error was made in care	1.14	0.624	0.68	1.89	Lao PDR	LMI
Aged 50+	1.41	0.020	1.05	1.88	Lao PDR	LMI
Has a chronic illness	0.75	0.048	0.57	1.00	Lao PDR	LMI
Had COVID	1.02	0.921	0.75	1.37	Lao PDR	LMI
Attended post-secondary education	1.67	0.000	1.28	2.18	Lao PDR	LMI
Highest income group	1.43	0.009	1.09	1.87	Lao PDR	LMI
Female	1.16	0.255	0.90	1.49	Lao PDR	LMI
Urban	1.47	0.006	1.12	1.92	Lao PDR	LMI
Minority ethnic or racial group	0.75	0.070	0.56	1.02	Lao PDR	LMI
_cons	0.69	0.020	0.51	0.94	Lao PDR	LMI
Rates usual facility/provider quality as very good or excellent	1.22	0.141	0.94	1.59	Kenya	LMI
Experienced discrimination in health system	1.02	0.930	0.65	1.61	Kenya	LMI
Believes medical error was made in care	0.70	0.121	0.44	1.10	Kenya	LMI
Aged 50+	1.92	0.001	1.28	2.86	Kenya	LMI
Has a chronic illness	1.17	0.403	0.81	1.69	Kenya	LMI
Had COVID	1.15	0.641	0.64	2.04	Kenya	LMI
Attended post-secondary education	1.93	0.000	1.43	2.60	Kenya	LMI
Highest income group	1.41	0.023	1.05	1.89	Kenya	LMI
Female	1.01	0.955	0.78	1.31	Kenya	LMI
Urban	1.02	0.875	0.77	1.37	Kenya	LMI
Minority ethnic or racial group	0.88	0.373	0.66	1.17	Kenya	LMI
_cons	0.68	0.016	0.50	0.93	Kenya	LMI
Rates usual facility/provider quality as very good or excellent	1.63	0.060	0.98	2.73	Italy	HI
Experienced discrimination in health system	0.54	0.328	0.16	1.85	Italy	HI
Believes medical error was made in care	1.49	0.485	0.49	4.54	Italy	HI
Aged 50+	3.84	0.000	2.21	6.68	Italy	HI
Has a chronic illness	1.22	0.479	0.70	2.12	Italy	HI
Had COVID	0.24	0.000	0.13	0.46	Italy	HI
Attended post-secondary education	1.12	0.683	0.65	1.95	Italy	HI
Highest income group	1.91	0.052	1.00	3.65	Italy	HI
Female	0.97	0.896	0.57	1.64	Italy	HI
Urban	1.68	0.481	0.40	7.08	Italy	HI
_cons	2.82	0.209	0.56	14.15	Italy	HI
Rates usual facility/provider quality as very good or excellent	1.14	0.455	0.81	1.60	Uruguay	HI
Experienced discrimination in health system	0.44	0.003	0.25	0.76	Uruguay	HI
Believes medical error was made in care	0.80	0.401	0.47	1.35	Uruguay	HI
Aged 50+	1.95	0.001	1.33	2.87	Uruguay	HI
Has a chronic illness	1.61	0.010	1.12	2.33	Uruguay	HI
Had COVID	0.74	0.075	0.53	1.03	Uruguay	HI
Attended post-secondary education	1.63	0.026	1.06	2.52	Uruguay	HI
Highest income group	2.10	0.000	1.39	3.18	Uruguay	HI
Female	1.21	0.288	0.85	1.71	Uruguay	HI
Urban	0.40	0.047	0.16	0.99	Uruguay	HI

	aOR	p-value	LCL	UCL	Country	Income group
_cons	4.79	0.002	1.81	12.73	Uruguay	HI
Rates usual facility/provider quality as very good or excellent	0.93	0.584	0.73	1.20	Korea	HI
Experienced discrimination in health system	0.90	0.727	0.51	1.60	Korea	HI
Believes medical error was made in care	0.44	0.001	0.28	0.70	Korea	HI
Aged 50+	2.90	0.000	2.23	3.75	Korea	HI
Has a chronic illness	1.31	0.037	1.02	1.69	Korea	HI
Had COVID	0.91	0.453	0.70	1.17	Korea	HI
Attended post-secondary education	1.18	0.229	0.90	1.56	Korea	HI
Highest income group	0.90	0.435	0.69	1.17	Korea	HI
Female	0.72	0.009	0.56	0.92	Korea	HI
Urban	0.94	0.753	0.65	1.37	Korea	HI
_cons	1.27	0.341	0.78	2.09	Korea	HI
Rates usual facility/provider quality as very good or excellent	0.90	0.649	0.57	1.42	India	LMI
Experienced discrimination in health system	0.71	0.566	0.22	2.27	India	LMI
Believes medical error was made in care	0.88	0.792	0.33	2.35	India	LMI
Aged 50+	2.92	0.008	1.32	6.46	India	LMI
Has a chronic illness	1.20	0.542	0.66	2.19	India	LMI
Had COVID	1.20	0.669	0.52	2.79	India	LMI
Attended post-secondary education	0.86	0.490	0.56	1.33	India	LMI
Highest income group	2.03	0.042	1.02	4.02	India	LMI
Female	0.62	0.033	0.40	0.96	India	LMI
Urban	0.83	0.385	0.54	1.27	India	LMI
_cons	0.44	0.000	0.29	0.68	India	LMI
Rates usual facility/provider quality as very good or excellent	0.99	0.938	0.72	1.36	Colombia	UMI
Experienced discrimination in health system	0.66	0.202	0.35	1.25	Colombia	UMI
Believes medical error was made in care	0.71	0.163	0.44	1.15	Colombia	UMI
Aged 50+	4.36	0.000	3.04	6.26	Colombia	UMI
Has a chronic illness	1.21	0.305	0.84	1.72	Colombia	UMI
Had COVID	0.98	0.912	0.71	1.37	Colombia	UMI
Attended post-secondary education	1.69	0.004	1.19	2.42	Colombia	UMI
Highest income group	1.57	0.009	1.12	2.20	Colombia	UMI
Female	0.97	0.863	0.71	1.33	Colombia	UMI
Urban	2.53	0.001	1.47	4.34	Colombia	UMI
_cons	0.12	0.000	0.06	0.21	Colombia	UMI
Rates usual facility/provider quality as very good or excellent	1.22	0.232	0.88	1.70	Argentina	UMI
Experienced discrimination in health system	0.57	0.019	0.35	0.91	Argentina	UMI
Believes medical error was made in care	0.92	0.754	0.56	1.53	Argentina	UMI
Aged 50+	3.29	0.000	2.30	4.70	Argentina	UMI
Has a chronic illness	1.45	0.038	1.02	2.05	Argentina	UMI
Had COVID	1.05	0.768	0.76	1.45	Argentina	UMI
Attended post-secondary education	1.54	0.024	1.06	2.23	Argentina	UMI
Highest income group	1.14	0.550	0.74	1.75	Argentina	UMI
Female	1.05	0.777	0.75	1.47	Argentina	UMI
Urban	1.25	0.527	0.63	2.46	Argentina	UMI
_cons	0.84	0.648	0.40	1.76	Argentina	UMI
Rates usual facility/provider quality as very good or excellent	1.05	0.654	0.85	1.30	Ethiopia	LMI
Experienced discrimination in health system	0.87	0.436	0.60	1.24	Ethiopia	LMI
Believes medical error was made in care	0.75	0.195	0.48	1.16	Ethiopia	LMI
Aged 50+	1.19	0.262	0.88	1.60	Ethiopia	LMI
Has a chronic illness	1.24	0.108	0.95	1.60	Ethiopia	LMI
Had COVID	1.24	0.508	0.65	2.37	Ethiopia	LMI
Attended post-secondary education	1.17	0.213	0.91	1.49	Ethiopia	LMI
Highest income group	0.95	0.702	0.75	1.21	Ethiopia	LMI
Female	0.92	0.452	0.74	1.14	Ethiopia	LMI
Urban	0.73	0.014	0.57	0.94	Ethiopia	LMI

	aOR	p-value	LCL	UCL	Country	Income group
Minority ethnic or racial group	2.17	0.000	1.70	2.76	Ethiopia	LMI
_cons	0.58	0.000	0.44	0.76	Ethiopia	LMI

aOR is adjusted Odds Ratio. LCL and UCL are lower- and upper confidence limits of the 95% confidence interval. LMI are low- and lower-middle-income countries. UMI are upper-middle-income countries. HI are high-income countries. _cons represents the baseline odds. Regressions are performed separately in each country.

Supplemental table 8. Model 4 Health security – Country-specific regression results for the odds of COVID vaccination (2+ or 3+ doses)

	aOR	p-value	LCL	UCL	Country	Income group
Confident can get and afford quality care	1.65	0.001	1.24	2.18	UK	HI
Aged 50+	7.13	0.000	4.98	10.19	UK	HI
Has a chronic illness	1.42	0.014	1.07	1.89	UK	HI
Had COVID	0.94	0.698	0.69	1.28	UK	HI
Attended post-secondary education	1.84	0.000	1.31	2.57	UK	HI
Highest income group	1.78	0.000	1.31	2.41	UK	HI
Female	0.80	0.128	0.61	1.06	UK	HI
Urban	1.35	0.213	0.84	2.16	UK	HI
Minority ethnic or racial group	0.59	0.016	0.39	0.91	UK	HI
_cons	0.62	0.136	0.33	1.16	UK	HI
Confident can get and afford quality care	1.55	0.000	1.23	1.95	USA	HI
Aged 50+	2.34	0.000	1.83	2.98	USA	HI
Has a chronic illness	1.34	0.012	1.07	1.69	USA	HI
Had COVID	0.75	0.012	0.60	0.94	USA	HI
Attended post-secondary education	2.53	0.000	1.96	3.25	USA	HI
Highest income group	1.64	0.000	1.29	2.08	USA	HI
Female	1.01	0.915	0.81	1.27	USA	HI
Urban	1.64	0.007	1.14	2.35	USA	HI
Minority ethnic or racial group	1.14	0.288	0.90	1.45	USA	HI
_cons	0.13	0.000	0.08	0.22	USA	HI
Confident can get and afford quality care	1.13	0.201	0.94	1.37	South Africa	UMI
Aged 50+	2.23	0.000	1.66	2.99	South Africa	UMI
Has a chronic illness	1.84	0.000	1.45	2.33	South Africa	UMI
Had COVID	1.85	0.000	1.40	2.45	South Africa	UMI
Attended post-secondary education	1.23	0.060	0.99	1.52	South Africa	UMI
Highest income group	1.29	0.019	1.04	1.59	South Africa	UMI
Female	1.40	0.001	1.15	1.69	South Africa	UMI
Urban	1.57	0.000	1.24	1.99	South Africa	UMI
Minority ethnic or racial group	0.91	0.398	0.73	1.13	South Africa	UMI
_cons	0.23	0.000	0.17	0.30	South Africa	UMI
Confident can get and afford quality care	0.98	0.915	0.64	1.48	Peru	UMI
Aged 50+	1.40	0.183	0.85	2.30	Peru	UMI
Has a chronic illness	0.90	0.667	0.56	1.44	Peru	UMI
Had COVID	2.00	0.000	1.36	2.94	Peru	UMI
Attended post-secondary education	2.67	0.000	1.69	4.24	Peru	UMI
Highest income group	1.89	0.038	1.04	3.44	Peru	UMI
Female	1.32	0.132	0.92	1.89	Peru	UMI
Urban	1.01	0.973	0.56	1.81	Peru	UMI
Minority ethnic or racial group	0.74	0.286	0.43	1.29	Peru	UMI
_cons	3.39	0.000	1.78	6.48	Peru	UMI
Confident can get and afford quality care	1.01	0.931	0.76	1.36	Mexico	UMI
Aged 50+	2.44	0.000	1.77	3.37	Mexico	UMI
Has a chronic illness	1.18	0.342	0.84	1.68	Mexico	UMI
Had COVID	1.34	0.057	0.99	1.81	Mexico	UMI
Attended post-secondary education	1.42	0.038	1.02	1.98	Mexico	UMI
Highest income group	1.04	0.815	0.73	1.50	Mexico	UMI
Female	1.10	0.533	0.82	1.48	Mexico	UMI
Urban	1.14	0.480	0.79	1.64	Mexico	UMI
Minority ethnic or racial group	0.74	0.260	0.43	1.25	Mexico	UMI
_cons	0.69	0.114	0.44	1.09	Mexico	UMI
Confident can get and afford quality care	1.35	0.007	1.09	1.68	Lao PDR	LMI
Aged 50+	1.47	0.001	1.17	1.84	Lao PDR	LMI

	aOR	p-value	LCL	UCL	Country	Income group
Has a chronic illness	0.73	0.006	0.58	0.91	Lao PDR	LMI
Had COVID	0.98	0.896	0.77	1.25	Lao PDR	LMI
Attended post-secondary education	1.71	0.000	1.39	2.10	Lao PDR	LMI
Highest income group	1.28	0.021	1.04	1.57	Lao PDR	LMI
Female	1.14	0.195	0.93	1.39	Lao PDR	LMI
Urban	1.33	0.009	1.07	1.64	Lao PDR	LMI
Minority ethnic or racial group	0.65	0.000	0.51	0.82	Lao PDR	LMI
_cons	0.63	0.001	0.47	0.83	Lao PDR	LMI
Confident can get and afford quality care	1.05	0.617	0.88	1.25	Kenya	LMI
Aged 50+	2.08	0.000	1.53	2.81	Kenya	LMI
Has a chronic illness	1.21	0.195	0.91	1.62	Kenya	LMI
Had COVID	1.26	0.300	0.82	1.93	Kenya	LMI
Attended post-secondary education	1.63	0.000	1.33	2.00	Kenya	LMI
Highest income group	1.37	0.002	1.12	1.66	Kenya	LMI
Female	1.26	0.011	1.05	1.52	Kenya	LMI
Urban	0.95	0.632	0.79	1.16	Kenya	LMI
Minority ethnic or racial group	0.92	0.401	0.75	1.12	Kenya	LMI
_cons	0.62	0.000	0.50	0.77	Kenya	LMI
Confident can get and afford quality care	1.87	0.001	1.29	2.72	Italy	HI
Aged 50+	2.90	0.000	1.97	4.28	Italy	HI
Has a chronic illness	1.27	0.266	0.84	1.92	Italy	HI
Had COVID	0.35	0.000	0.23	0.53	Italy	HI
Attended post-secondary education	1.18	0.432	0.78	1.76	Italy	HI
Highest income group	1.44	0.105	0.93	2.23	Italy	HI
Female	0.97	0.885	0.67	1.42	Italy	HI
Urban	2.08	0.096	0.88	4.92	Italy	HI
_cons	1.39	0.501	0.53	3.61	Italy	HI
Confident can get and afford quality care	1.64	0.002	1.19	2.26	Uruguay	HI
Aged 50+	2.23	0.000	1.59	3.13	Uruguay	HI
Has a chronic illness	1.43	0.029	1.04	1.98	Uruguay	HI
Had COVID	0.78	0.106	0.58	1.05	Uruguay	HI
Attended post-secondary education	1.57	0.018	1.08	2.28	Uruguay	HI
Highest income group	1.91	0.000	1.34	2.73	Uruguay	HI
Female	1.22	0.207	0.90	1.65	Uruguay	HI
Urban	0.71	0.321	0.36	1.39	Uruguay	HI
_cons	2.04	0.044	1.02	4.08	Uruguay	HI
Confident can get and afford quality care	1.04	0.670	0.86	1.26	Korea	HI
Aged 50+	2.91	0.000	2.39	3.55	Korea	HI
Has a chronic illness	1.18	0.105	0.97	1.44	Korea	HI
Had COVID	0.92	0.422	0.76	1.12	Korea	HI
Attended post-secondary education	1.14	0.208	0.93	1.41	Korea	HI
Highest income group	1.07	0.517	0.87	1.32	Korea	HI
Female	0.81	0.032	0.67	0.98	Korea	HI
Urban	1.05	0.721	0.80	1.39	Korea	HI
_cons	0.91	0.608	0.63	1.31	Korea	HI
Confident can get and afford quality care	1.34	0.025	1.04	1.73	India	LMI
Aged 50+	1.64	0.027	1.06	2.55	India	LMI
Has a chronic illness	1.43	0.072	0.97	2.11	India	LMI
Had COVID	0.99	0.974	0.57	1.72	India	LMI
Attended post-secondary education	0.95	0.654	0.74	1.20	India	LMI
Highest income group	1.58	0.008	1.13	2.21	India	LMI
Female	0.82	0.109	0.65	1.04	India	LMI
Urban	0.98	0.866	0.78	1.24	India	LMI
_cons	0.29	0.000	0.22	0.40	India	LMI
Confident can get and afford quality care	1.40	0.018	1.06	1.85	Colombia	UMI

	aOR	p-value	LCL	UCL	Country	Income group
Aged 50+	4.25	0.000	3.14	5.74	Colombia	UMI
Has a chronic illness	1.18	0.298	0.86	1.61	Colombia	UMI
Had COVID	0.99	0.930	0.75	1.31	Colombia	UMI
Attended post-secondary education	1.73	0.000	1.28	2.33	Colombia	UMI
Highest income group	1.48	0.007	1.11	1.98	Colombia	UMI
Female	1.26	0.088	0.97	1.64	Colombia	UMI
Urban	2.02	0.002	1.28	3.18	Colombia	UMI
_cons	0.10	0.000	0.06	0.16	Colombia	UMI
Confident can get and afford quality care	1.15	0.371	0.85	1.55	Argentina	UMI
Aged 50+	3.01	0.000	2.23	4.06	Argentina	UMI
Has a chronic illness	1.30	0.079	0.97	1.75	Argentina	UMI
Had COVID	0.94	0.635	0.71	1.23	Argentina	UMI
Attended post-secondary education	1.41	0.029	1.04	1.92	Argentina	UMI
Highest income group	1.36	0.105	0.94	1.96	Argentina	UMI
Female	1.20	0.208	0.90	1.58	Argentina	UMI
Urban	0.98	0.946	0.55	1.74	Argentina	UMI
_cons	0.95	0.863	0.52	1.73	Argentina	UMI
Confident can get and afford quality care	1.35	0.000	1.14	1.59	Ethiopia	LMI
Aged 50+	1.13	0.297	0.89	1.44	Ethiopia	LMI
Has a chronic illness	1.26	0.043	1.01	1.58	Ethiopia	LMI
Had COVID	1.23	0.455	0.71	2.12	Ethiopia	LMI
Attended post-secondary education	1.20	0.069	0.99	1.45	Ethiopia	LMI
Highest income group	0.89	0.229	0.74	1.07	Ethiopia	LMI
Female	1.02	0.800	0.86	1.21	Ethiopia	LMI
Urban	0.73	0.002	0.60	0.89	Ethiopia	LMI
Minority ethnic or racial group	2.01	0.000	1.66	2.42	Ethiopia	LMI
_cons	0.46	0.000	0.37	0.58	Ethiopia	LMI

aOR is adjusted Odds Ratio. LCL and UCL are lower- and upper confidence limits of the 95% confidence interval. LMI are low- and lower-middle-income countries. UMI are upper-middle-income countries. HI are high-income countries. _cons represents the baseline odds. Regressions are performed separately in each country.

Supplemental table 9. Model 5 Government responsiveness to public opinion – Country-specific regression results for the odds of COVID vaccination (2+ or 3+ doses)

	aOR	p-value	LCL	UCL	Country	Income group
Confident government considers public opinion in health	1.11	0.526	0.81	1.51	UK	HI
Aged 50+	7.05	0.000	4.97	10.01	UK	HI
Has a chronic illness	1.40	0.017	1.06	1.85	UK	HI
Had COVID	0.92	0.601	0.68	1.25	UK	HI
Attended post-secondary education	1.89	0.000	1.37	2.63	UK	HI
Highest income group	1.93	0.000	1.43	2.60	UK	HI
Female	0.81	0.125	0.61	1.06	UK	HI
Urban	1.35	0.195	0.86	2.13	UK	HI
Minority ethnic or racial group	0.56	0.007	0.37	0.85	UK	HI
_cons	0.73	0.319	0.40	1.35	UK	HI
Confident government considers public opinion in health	1.58	0.000	1.25	1.99	USA	HI
Aged 50+	2.46	0.000	1.94	3.12	USA	HI
Has a chronic illness	1.35	0.011	1.07	1.70	USA	HI
Had COVID	0.75	0.014	0.60	0.94	USA	HI
Attended post-secondary education	2.54	0.000	1.97	3.28	USA	HI
Highest income group	1.77	0.000	1.39	2.24	USA	HI
Female	1.02	0.897	0.81	1.28	USA	HI
Urban	1.64	0.007	1.14	2.35	USA	HI
Minority ethnic or racial group	1.06	0.612	0.84	1.36	USA	HI
_cons	0.14	0.000	0.09	0.23	USA	HI
Confident government considers public opinion in health	1.00	0.992	0.82	1.21	South Africa	UMI
Aged 50+	2.19	0.000	1.64	2.94	South Africa	UMI
Has a chronic illness	1.83	0.000	1.44	2.32	South Africa	UMI
Had COVID	1.81	0.000	1.36	2.39	South Africa	UMI
Attended post-secondary education	1.21	0.089	0.97	1.49	South Africa	UMI
Highest income group	1.30	0.013	1.06	1.60	South Africa	UMI
Female	1.40	0.001	1.15	1.70	South Africa	UMI
Urban	1.55	0.000	1.22	1.96	South Africa	UMI
Minority ethnic or racial group	0.91	0.404	0.73	1.13	South Africa	UMI
_cons	0.24	0.000	0.18	0.32	South Africa	UMI
Confident government considers public opinion in health	1.25	0.254	0.85	1.81	Peru	UMI
Aged 50+	1.45	0.143	0.88	2.39	Peru	UMI
Has a chronic illness	0.93	0.770	0.58	1.49	Peru	UMI
Had COVID	1.99	0.001	1.35	2.92	Peru	UMI
Attended post-secondary education	2.74	0.000	1.74	4.32	Peru	UMI
Highest income group	1.92	0.031	1.06	3.48	Peru	UMI
Female	1.29	0.160	0.90	1.86	Peru	UMI
Urban	1.01	0.969	0.57	1.81	Peru	UMI
Minority ethnic or racial group	0.75	0.304	0.43	1.30	Peru	UMI
_cons	3.05	0.001	1.58	5.89	Peru	UMI
Confident government considers public opinion in health	1.09	0.572	0.80	1.49	Mexico	UMI
Aged 50+	2.37	0.000	1.72	3.27	Mexico	UMI
Has a chronic illness	1.20	0.301	0.85	1.70	Mexico	UMI
Had COVID	1.37	0.038	1.02	1.85	Mexico	UMI
Attended post-secondary education	1.42	0.039	1.02	1.98	Mexico	UMI
Highest income group	1.04	0.827	0.73	1.49	Mexico	UMI
Female	1.07	0.640	0.80	1.44	Mexico	UMI
Urban	1.15	0.446	0.80	1.64	Mexico	UMI
Minority ethnic or racial group	0.71	0.213	0.42	1.22	Mexico	UMI
_cons	0.66	0.083	0.41	1.06	Mexico	UMI

	aOR	p-value	LCL	UCL	Country	Income group
Confident government considers public opinion in health	1.36	0.010	1.08	1.72	Lao PDR	LMI
Aged 50+	1.47	0.001	1.17	1.84	Lao PDR	LMI
Has a chronic illness	0.72	0.005	0.57	0.90	Lao PDR	LMI
Had COVID	0.99	0.961	0.78	1.27	Lao PDR	LMI
Attended post-secondary education	1.70	0.000	1.38	2.10	Lao PDR	LMI
Highest income group	1.29	0.018	1.04	1.59	Lao PDR	LMI
Female	1.15	0.159	0.95	1.41	Lao PDR	LMI
Urban	1.38	0.003	1.12	1.71	Lao PDR	LMI
Minority ethnic or racial group	0.65	0.000	0.52	0.83	Lao PDR	LMI
_cons	0.61	0.001	0.44	0.82	Lao PDR	LMI
Confident government considers public opinion in health	0.77	0.004	0.64	0.92	Kenya	LMI
Aged 50+	2.03	0.000	1.50	2.75	Kenya	LMI
Has a chronic illness	1.19	0.230	0.89	1.60	Kenya	LMI
Had COVID	1.22	0.365	0.79	1.88	Kenya	LMI
Attended post-secondary education	1.58	0.000	1.29	1.94	Kenya	LMI
Highest income group	1.39	0.001	1.14	1.69	Kenya	LMI
Female	1.28	0.008	1.07	1.54	Kenya	LMI
Urban	0.94	0.536	0.77	1.14	Kenya	LMI
Minority ethnic or racial group	0.92	0.396	0.75	1.12	Kenya	LMI
_cons	0.75	0.012	0.60	0.94	Kenya	LMI
Confident government considers public opinion in health	1.15	0.457	0.79	1.67	Italy	HI
Aged 50+	2.81	0.000	1.91	4.13	Italy	HI
Has a chronic illness	1.29	0.227	0.85	1.95	Italy	HI
Had COVID	0.35	0.000	0.23	0.53	Italy	HI
Attended post-secondary education	1.20	0.376	0.80	1.81	Italy	HI
Highest income group	1.47	0.081	0.95	2.27	Italy	HI
Female	0.95	0.809	0.66	1.39	Italy	HI
Urban	2.02	0.123	0.83	4.92	Italy	HI
_cons	1.99	0.170	0.75	5.30	Italy	HI
Confident government considers public opinion in health	1.66	0.002	1.20	2.29	Uruguay	HI
Aged 50+	2.20	0.000	1.56	3.09	Uruguay	HI
Has a chronic illness	1.48	0.018	1.07	2.04	Uruguay	HI
Had COVID	0.80	0.141	0.59	1.08	Uruguay	HI
Attended post-secondary education	1.58	0.018	1.08	2.30	Uruguay	HI
Highest income group	2.11	0.000	1.47	3.01	Uruguay	HI
Female	1.30	0.092	0.96	1.76	Uruguay	HI
Urban	0.73	0.361	0.37	1.43	Uruguay	HI
_cons	1.84	0.089	0.91	3.73	Uruguay	HI
Confident government considers public opinion in health	0.94	0.539	0.78	1.14	Korea	HI
Aged 50+	2.92	0.000	2.40	3.55	Korea	HI
Has a chronic illness	1.17	0.111	0.96	1.43	Korea	HI
Had COVID	0.92	0.415	0.76	1.12	Korea	HI
Attended post-secondary education	1.15	0.201	0.93	1.41	Korea	HI
Highest income group	1.08	0.474	0.88	1.32	Korea	HI
Female	0.80	0.025	0.66	0.97	Korea	HI
Urban	1.05	0.714	0.80	1.39	Korea	HI
_cons	0.96	0.843	0.67	1.38	Korea	HI
Confident government considers public opinion in health	1.29	0.071	0.98	1.71	India	LMI
Aged 50+	1.56	0.041	1.02	2.41	India	LMI
Has a chronic illness	1.36	0.129	0.91	2.03	India	LMI
Had COVID	0.99	0.977	0.58	1.71	India	LMI
Attended post-secondary education	0.95	0.695	0.75	1.21	India	LMI
Highest income group	1.58	0.008	1.12	2.21	India	LMI
Female	0.80	0.063	0.63	1.01	India	LMI
Urban	1.01	0.940	0.80	1.28	India	LMI

	aOR	p-value	LCL	UCL	Country	Income group
_cons	0.29	0.000	0.21	0.40	India	LMI
Confident government considers public opinion in health	1.21	0.165	0.93	1.57	Colombia	UMI
Aged 50+	4.50	0.000	3.33	6.07	Colombia	UMI
Has a chronic illness	1.13	0.441	0.83	1.54	Colombia	UMI
Had COVID	0.97	0.848	0.74	1.29	Colombia	UMI
Attended post-secondary education	1.69	0.001	1.26	2.29	Colombia	UMI
Highest income group	1.47	0.008	1.11	1.95	Colombia	UMI
Female	1.26	0.083	0.97	1.64	Colombia	UMI
Urban	2.02	0.002	1.30	3.14	Colombia	UMI
_cons	0.10	0.000	0.06	0.17	Colombia	UMI
Confident government considers public opinion in health	1.06	0.732	0.78	1.44	Argentina	UMI
Aged 50+	3.01	0.000	2.23	4.06	Argentina	UMI
Has a chronic illness	1.27	0.113	0.94	1.70	Argentina	UMI
Had COVID	0.97	0.850	0.74	1.28	Argentina	UMI
Attended post-secondary education	1.44	0.019	1.06	1.96	Argentina	UMI
Highest income group	1.43	0.053	1.00	2.05	Argentina	UMI
Female	1.22	0.160	0.92	1.62	Argentina	UMI
Urban	1.03	0.917	0.58	1.82	Argentina	UMI
_cons	0.89	0.712	0.49	1.62	Argentina	UMI
Confident government considers public opinion in health	1.95	0.000	1.58	2.40	Ethiopia	LMI
Aged 50+	1.13	0.317	0.89	1.44	Ethiopia	LMI
Has a chronic illness	1.27	0.041	1.01	1.59	Ethiopia	LMI
Had COVID	1.24	0.433	0.72	2.13	Ethiopia	LMI
Attended post-secondary education	1.20	0.072	0.98	1.45	Ethiopia	LMI
Highest income group	0.91	0.300	0.75	1.09	Ethiopia	LMI
Female	1.01	0.899	0.85	1.20	Ethiopia	LMI
Urban	0.76	0.005	0.62	0.92	Ethiopia	LMI
Minority ethnic or racial group	1.99	0.000	1.65	2.40	Ethiopia	LMI
_cons	0.32	0.000	0.24	0.41	Ethiopia	LMI

Supplemental table 10. Model 6 Government management of the COVID-19 pandemic – Country-specific regression results for the odds of COVID vaccination (2+ or 3+ doses)

	aOR	p-value	LCL	UCL	Country	Income group
Rates government management of COVID very good or excellent	1.82	0.002	1.24	2.67	UK	HI
Aged 50+	6.57	0.000	4.61	9.38	UK	HI
Has a chronic illness	1.42	0.014	1.07	1.88	UK	HI
Had COVID	0.93	0.623	0.68	1.26	UK	HI
Attended post-secondary education	2.01	0.000	1.44	2.81	UK	HI
Highest income group	1.85	0.000	1.37	2.50	UK	HI
Female	0.82	0.170	0.63	1.09	UK	HI
Urban	1.40	0.144	0.89	2.21	UK	HI
Minority ethnic or racial group	0.56	0.009	0.37	0.86	UK	HI
_cons	0.65	0.165	0.35	1.20	UK	HI
Rates government management of COVID very good or excellent	3.37	0.000	2.51	4.54	USA	HI
Aged 50+	2.14	0.000	1.67	2.74	USA	HI
Has a chronic illness	1.30	0.027	1.03	1.65	USA	HI
Had COVID	0.77	0.027	0.61	0.97	USA	HI
Attended post-secondary education	2.61	0.000	2.02	3.39	USA	HI
Highest income group	1.72	0.000	1.34	2.19	USA	HI
Female	1.08	0.510	0.86	1.37	USA	HI
Urban	1.58	0.012	1.10	2.25	USA	HI
Minority ethnic or racial group	0.97	0.782	0.75	1.24	USA	HI
_cons	0.14	0.000	0.09	0.23	USA	HI
Rates government management of COVID very good or excellent	1.37	0.002	1.12	1.67	South Africa	UMI
Aged 50+	2.29	0.000	1.70	3.10	South Africa	UMI
Has a chronic illness	1.81	0.000	1.43	2.31	South Africa	UMI
Had COVID	1.82	0.000	1.37	2.41	South Africa	UMI
Attended post-secondary education	1.25	0.043	1.01	1.55	South Africa	UMI
Highest income group	1.35	0.006	1.09	1.66	South Africa	UMI
Female	1.41	0.000	1.16	1.72	South Africa	UMI
Urban	1.58	0.000	1.25	2.01	South Africa	UMI
Minority ethnic or racial group	0.89	0.292	0.71	1.11	South Africa	UMI
_cons	0.21	0.000	0.16	0.27	South Africa	UMI
Rates government management of COVID very good or excellent	1.04	0.891	0.57	1.91	Peru	UMI
Aged 50+	1.50	0.118	0.90	2.48	Peru	UMI
Has a chronic illness	0.89	0.643	0.56	1.43	Peru	UMI
Had COVID	1.92	0.001	1.31	2.81	Peru	UMI
Attended post-secondary education	2.89	0.000	1.80	4.63	Peru	UMI
Highest income group	1.98	0.030	1.07	3.68	Peru	UMI
Female	1.26	0.214	0.88	1.81	Peru	UMI
Urban	1.03	0.918	0.58	1.84	Peru	UMI
Minority ethnic or racial group	0.80	0.432	0.46	1.40	Peru	UMI
_cons	3.32	0.000	1.76	6.27	Peru	UMI
Rates government management of COVID very good or excellent	1.12	0.490	0.81	1.56	Mexico	UMI
Aged 50+	2.39	0.000	1.74	3.28	Mexico	UMI
Has a chronic illness	1.18	0.342	0.84	1.67	Mexico	UMI
Had COVID	1.39	0.029	1.03	1.87	Mexico	UMI
Attended post-secondary education	1.39	0.053	1.00	1.93	Mexico	UMI
Highest income group	1.05	0.791	0.74	1.50	Mexico	UMI
Female	1.08	0.618	0.80	1.45	Mexico	UMI
Urban	1.16	0.410	0.81	1.66	Mexico	UMI
Minority ethnic or racial group	0.74	0.281	0.43	1.27	Mexico	UMI

	aOR	p-value	LCL	UCL	Country	Income group
_cons	0.68	0.064	0.45	1.02	Mexico	UMI
Rates government management of COVID very good or excellent	1.12	0.273	0.92	1.36	Lao PDR	LMI
Aged 50+	1.48	0.001	1.18	1.85	Lao PDR	LMI
Has a chronic illness	0.72	0.004	0.57	0.90	Lao PDR	LMI
Had COVID	0.99	0.965	0.78	1.27	Lao PDR	LMI
Attended post-secondary education	1.71	0.000	1.39	2.11	Lao PDR	LMI
Highest income group	1.31	0.011	1.06	1.61	Lao PDR	LMI
Female	1.17	0.129	0.96	1.42	Lao PDR	LMI
Urban	1.32	0.010	1.07	1.63	Lao PDR	LMI
Minority ethnic or racial group	0.65	0.000	0.52	0.82	Lao PDR	LMI
_cons	0.73	0.012	0.57	0.93	Lao PDR	LMI
Rates government management of COVID very good or excellent	0.99	0.954	0.83	1.19	Kenya	LMI
Aged 50+	2.11	0.000	1.56	2.86	Kenya	LMI
Has a chronic illness	1.23	0.161	0.92	1.65	Kenya	LMI
Had COVID	1.25	0.310	0.81	1.93	Kenya	LMI
Attended post-secondary education	1.64	0.000	1.34	2.01	Kenya	LMI
Highest income group	1.37	0.002	1.13	1.66	Kenya	LMI
Female	1.28	0.008	1.07	1.53	Kenya	LMI
Urban	0.95	0.600	0.78	1.15	Kenya	LMI
Minority ethnic or racial group	0.91	0.379	0.74	1.12	Kenya	LMI
_cons	0.64	0.000	0.51	0.79	Kenya	LMI
Rates government management of COVID very good or excellent	1.73	0.015	1.11	2.68	Italy	HI
Aged 50+	2.89	0.000	1.97	4.24	Italy	HI
Has a chronic illness	1.20	0.387	0.80	1.80	Italy	HI
Had COVID	0.33	0.000	0.22	0.50	Italy	HI
Attended post-secondary education	1.16	0.493	0.76	1.75	Italy	HI
Highest income group	1.47	0.085	0.95	2.28	Italy	HI
Female	0.97	0.866	0.67	1.41	Italy	HI
Urban	1.85	0.177	0.76	4.54	Italy	HI
_cons	2.13	0.126	0.81	5.58	Italy	HI
Rates government management of COVID very good or excellent	2.95	0.000	2.17	4.01	Uruguay	HI
Aged 50+	2.03	0.000	1.44	2.86	Uruguay	HI
Has a chronic illness	1.60	0.005	1.15	2.22	Uruguay	HI
Had COVID	0.84	0.256	0.62	1.13	Uruguay	HI
Attended post-secondary education	1.58	0.018	1.08	2.32	Uruguay	HI
Highest income group	1.92	0.001	1.33	2.76	Uruguay	HI
Female	1.30	0.094	0.96	1.77	Uruguay	HI
Urban	0.81	0.556	0.41	1.62	Uruguay	HI
_cons	1.17	0.675	0.56	2.45	Uruguay	HI
Rates government management of COVID very good or excellent	1.29	0.018	1.04	1.58	Korea	HI
Aged 50+	2.84	0.000	2.33	3.47	Korea	HI
Has a chronic illness	1.18	0.106	0.97	1.44	Korea	HI
Had COVID	0.92	0.429	0.76	1.12	Korea	HI
Attended post-secondary education	1.14	0.225	0.92	1.40	Korea	HI
Highest income group	1.07	0.495	0.87	1.32	Korea	HI
Female	0.82	0.040	0.68	0.99	Korea	HI
Urban	1.05	0.743	0.79	1.39	Korea	HI
_cons	0.88	0.462	0.62	1.24	Korea	HI
Rates government management of COVID very good or excellent	0.77	0.029	0.61	0.97	India	LMI
Aged 50+	1.57	0.041	1.02	2.41	India	LMI
Has a chronic illness	1.36	0.121	0.92	2.01	India	LMI
Had COVID	1.09	0.775	0.62	1.90	India	LMI
Attended post-secondary education	0.94	0.591	0.74	1.19	India	LMI
Highest income group	1.62	0.004	1.17	2.26	India	LMI
Female	0.80	0.059	0.63	1.01	India	LMI

	aOR	p-value	LCL	UCL	Country	Income group
Urban	0.98	0.871	0.78	1.24	India	LMI
_cons	0.40	0.000	0.31	0.52	India	LMI
Rates government management of COVID very good or excellent	1.30	0.081	0.97	1.76	Colombia	UMI
Aged 50+	4.27	0.000	3.16	5.77	Colombia	UMI
Has a chronic illness	1.14	0.406	0.84	1.55	Colombia	UMI
Had COVID	0.96	0.761	0.72	1.27	Colombia	UMI
Attended post-secondary education	1.72	0.000	1.27	2.32	Colombia	UMI
Highest income group	1.46	0.009	1.10	1.94	Colombia	UMI
Female	1.24	0.111	0.95	1.61	Colombia	UMI
Urban	2.02	0.002	1.30	3.14	Colombia	UMI
_cons	0.11	0.000	0.07	0.17	Colombia	UMI
Rates government management of COVID very good or excellent	1.43	0.015	1.07	1.89	Argentina	UMI
Aged 50+	2.94	0.000	2.17	3.97	Argentina	UMI
Has a chronic illness	1.34	0.054	0.99	1.80	Argentina	UMI
Had COVID	0.96	0.791	0.73	1.27	Argentina	UMI
Attended post-secondary education	1.40	0.033	1.03	1.90	Argentina	UMI
Highest income group	1.45	0.043	1.01	2.08	Argentina	UMI
Female	1.20	0.205	0.91	1.58	Argentina	UMI
Urban	0.99	0.968	0.56	1.75	Argentina	UMI
_cons	0.85	0.602	0.47	1.55	Argentina	UMI
Rates government management of COVID very good or excellent	1.40	0.000	1.19	1.65	Ethiopia	LMI
Aged 50+	1.16	0.229	0.91	1.47	Ethiopia	LMI
Has a chronic illness	1.28	0.032	1.02	1.60	Ethiopia	LMI
Had COVID	1.10	0.723	0.64	1.91	Ethiopia	LMI
Attended post-secondary education	1.18	0.090	0.97	1.43	Ethiopia	LMI
Highest income group	0.88	0.178	0.73	1.06	Ethiopia	LMI
Female	1.01	0.919	0.85	1.20	Ethiopia	LMI
Urban	0.71	0.001	0.58	0.86	Ethiopia	LMI
Minority ethnic or racial group	1.84	0.000	1.52	2.22	Ethiopia	LMI
_cons	0.49	0.000	0.39	0.60	Ethiopia	LMI

Supplemental Table 11. Health system utilization and quality and COVID-19 vaccination with at least 2 doses, adjusted odds ratios pooled across countries and country income groups

	Low- and lower-middle income countries			Upper-middle income countries			High income countries			All countries		
	aOR	LCL	UCL	aOR	LCL	UCL	aOR	LCL	UCL	aOR	LCL	UCL
**Health care utilization**												
Had 1-2 visits in last year	1.11	0.87	1.41	1.69	1.34	2.12	2.59	1.95	3.45	1.67	1.33	2.10
Had 3-4 visits in last year	1.48	1.23	1.79	1.97	1.60	2.43	2.64	1.90	3.67	1.97	1.63	2.37
Had 5 or more visits in last year	1.31	1.09	1.59	2.72	1.80	4.12	3.33	2.10	5.29	2.26	1.70	3.00
**Health system competence**												
Has a usual source of care	1.16	0.89	1.51	1.35	1.15	1.58	1.29	1.05	1.58	1.24	1.10	1.41
Received at least 3 other preventive services in last year	1.54	1.32	1.81	1.40	1.14	1.72	1.76	1.45	2.13	1.54	1.37	1.73
Had unmet health care need in last year	0.80	0.64	0.99	0.75	0.60	0.93	0.52	0.37	0.73	0.69	0.59	0.81
**Perceived quality and user experience**												
Rated quality of usual provider very good or excellent	1.06	0.91	1.24	1.08	0.78	1.50	0.99	0.80	1.21	1.04	0.93	1.17
Experienced discrimination in the health system	0.73	0.50	1.07	0.69	0.52	0.91	0.62	0.43	0.89	0.71	0.61	0.84
Believes medical mistake was made in care	0.77	0.58	1.03	0.79	0.55	1.12	0.60	0.45	0.81	0.74	0.63	0.87
**Health security**												
Confident could get and afford quality care	1.23	1.06	1.43	1.11	0.96	1.28	1.67	1.34	2.08	1.31	1.16	1.47
**Government responsiveness to public opinion**												
Believes government considers public opinion	1.31	0.79	2.19	1.13	0.92	1.39	1.66	1.19	2.30	1.36	1.10	1.68
**Government management of the COVID-19 pandemic**												
Rates government's management of pandemic as very good or excellent	1.12	0.91	1.39	1.34	1.16	1.56	3.20	1.59	6.46	1.61	1.28	2.02

Low and lower-middle-income countries include Ethiopia, India, Kenya, and Lao PDR. Upper-middle-income countries include Argentina, Colombia, Mexico, Peru, and South Africa. High-income countries are Italy, Korea, Uruguay, the U.K. and the U.S.

aOR are adjusted odds ratio. LCL and UCL are lower and upper confidence limits. Estimates from six distinct country-specific regression models are pooled across countries and country income groups by inverse-variance-weighted random-effect meta-analysis. All underlying models were adjusted for age 50 and above, chronic illness, past COVID infection, post-secondary education, residing in an urban area, female, and highest income group. In Ethiopia, Kenya, Lao PDR, Mexico, Peru, South Africa and the USA, models also include whether the respondent belongs to a minority ethnic, racial, or linguistic group.

Supplemental Table 12. Health system utilization and quality and COVID-19 vaccination (2+ or 3+ doses), adjusted odds ratios pooled across countries groups by COVID severity

	Countries with fewer than 1000 COVID deaths per million			Countries with 1500-3000 COVID deaths per million			Countries with 3000-6500 COVID deaths per million		
	aOR	LCL	UCL	aOR	LCL	UCL	aOR	LCL	UCL
**Health care utilization**									
Had 1-2 visits in last year	1.03	0.85	1.25	1.62	1.25	2.09	1.71	1.33	2.21
Had 3-4 visits in last year	1.42	1.18	1.72	1.85	1.53	2.24	1.84	1.42	2.37
Had 5 or more visits in last year	1.31	0.99	1.72	2.14	1.55	2.97	2.31	1.79	2.99
**Health system competence**									
Has a usual source of care	1.10	0.97	1.26	1.29	1.11	1.49	1.35	1.10	1.66
Received at least 3 other preventive services in last year	1.40	1.20	1.63	1.59	1.34	1.88	1.75	1.47	2.10
Had unmet health care need in last year	0.82	0.66	1.02	0.73	0.57	0.93	0.71	0.59	0.86
**Perceived quality and user experience**									
Rated quality of usual provider very good or excellent	1.05	0.93	1.19	1.20	1.05	1.38	1.28	0.98	1.67
Experienced discrimination in the health system Believes	0.89	0.72	1.09	0.60	0.48	0.76	0.64	0.46	0.90
medical mistake was made in care	0.72	0.52	0.99	0.89	0.71	1.12	0.91	0.69	1.20
**Health security**									
Confident could get and afford quality care	1.21	1.06	1.37	1.23	1.05	1.44	1.51	1.21	1.88
**Government responsiveness to public opinion**									
Believes government considers public opinion	1.19	0.85	1.68	1.16	0.98	1.37	1.30	1.08	1.56
**Government management of the COVID-19 pandemic**									
Rates government's management of pandemic as very good or excellent	1.10	0.90	1.33	1.53	1.13	2.06	1.90	1.18	3.06

Countries with fewer than 1000 COVID deaths per million population at the start of the survey include Ethiopia, India, Kenya, South Korea, and Laos. Countries with 1500 to 3000 COVID deaths per million include South Africa, Uruguay, Mexico, Colombia, and Argentina. Countries with 3000 to 6500 COVID deaths per million include Italy, USA, UK, and Peru.

aOR are adjusted odds ratio. LCL and UCL are lower and upper confidence limits. Estimates from six distinct country-specific regression models are pooled across country groups by inverse-variance-weighted random-effect meta-analysis. All underlying models were adjusted for age 50 and above, chronic illness, past COVID infection, post-secondary education, residing in an urban area, female, and highest income group. In Ethiopia, Kenya, Lao PDR, Mexico, Peru, South Africa and the USA, models also include whether the respondent belongs to a minority ethnic, racial, or linguistic group.
